# Cholecystokinin-expressing neurons of the ventromedial hypothalamic nucleus control energy homeostasis

**DOI:** 10.3389/fncel.2024.1483368

**Published:** 2024-10-28

**Authors:** Vasileios Eftychidis, Tommas J. Ellender, Jacek Szymanski, Liliana Minichiello

**Affiliations:** ^1^Department of Pharmacology, University of Oxford, Oxford, United Kingdom; ^2^Department of Biomedical Sciences, University of Antwerp, Antwerp, Belgium

**Keywords:** hypothalamus, VMH, central CCK, food intake, body weight, glucose homeostasis, energy homeostasis

## Abstract

The hypothalamus is the primary center of the brain that regulates energy homeostasis. The ventromedial hypothalamus (VMH) plays a central role in maintaining energy balance by regulating food intake, energy expenditure, and glucose levels. However, the cellular and molecular mechanisms underlying its functions are still poorly understood. Cholecystokinin (CCK) is one of many genes expressed in this hypothalamic nucleus. Peripheral CCK regulates food intake, body weight, and glucose homeostasis. However, current research does not explain the function of CCK neurons in specific nuclei of the hypothalamus and their likely roles in network dynamics related to energy balance and food intake. This study uses genetic and pharmacological methods to examine the role of CCK-expressing neurons in the VMH (CCK^VMH^). Namely, using a previously generated BAC transgenic line expressing Cre recombinase under the CCK promoter, we performed targeted manipulations of CCK^VMH^ neurons. Histological and transcriptomic database analysis revealed extensive distribution of these neurons in the VMH, with significant heterogeneity in gene expression related to energy balance, including co-expression with PACAP and somatostatin. Pharmacogenetic acute inhibition via Designer Receptors Exclusively Activated by Designer Drugs (DREADDs) resulted in increased food intake and altered meal patterns, characterized by higher meal frequency and shorter intermeal intervals. Furthermore, diphtheria toxin-mediated ablation of CCK^VMH^ neurons led to significant weight gain and hyperphagia over time, increasing meal size and duration. These mice also exhibited impaired glucose tolerance, indicative of disrupted glucose homeostasis. Our findings underscore the integral role of CCK^VMH^ neurons in modulating feeding behavior, energy homeostasis, and glucose regulation. This study enhances our understanding of the neurohormonal mechanisms underlying obesity and metabolic disorders, providing potential targets for therapeutic interventions.

## 1 Introduction

The hypothalamus is a widely studied brain region. It is one of the most important regulators of food intake and energy homeostasis. It comprises several distinct nuclei, among which the ventromedial hypothalamic nucleus (VMH) was the first to be recognized as a site for body weight regulation and energy homeostasis (Hetherington and Ranson, 1940).

Over the years, the VMH has been associated with various physiological and behavioral functions (Hetherington and Ranson, 1940; King, 2006). The VMH, together with other hypothalamic nuclei, the lateral hypothalamus (LH), the dorsomedial hypothalamus (DMH), the arcuate nucleus of the hypothalamus (ARH), and the paraventricular hypothalamic nucleus (PVH), process incoming information and relay it to neuronal circuits beyond the hypothalamus, leading to an integrated response to food intake and energy expenditure (Kleinridders et al., 2009; Roh et al., 2016; Waterson and Horvath, 2015).

Recent studies using refined genetic tools have further highlighted the physiological role of the VMH in regulating energy and glucose homeostasis (Choi et al., 2013).

Like the rest of the hypothalamus, the VMH is highly heterogeneous in terms of cellular composition. It contains neurons responsible for sensing changes in glucose concentrations, as well as neurons equipped with receptors for various metabolic hormones (e.g., insulin and leptin) or neuropeptides associated with the regulation of energy balance (Affinati et al., 2023; Elmquist et al., 1997; Routh, 2003; Scott et al., 2009; Steuernagel et al., 2022).

The *Nr5a1* gene, which encodes steroidogenic factor 1 (SF1), is a transcription factor specifically expressed in the VMH within the adult rodent brain. However, it is also found in the adrenal gland, pituitary gonadotrophs, and gonads (Ikeda et al., 1995; Khodai and Luckman, 2021). *Nr5a1*-knockout mice were suggested as a genetic model for hypothalamic obesity. Interestingly, mice deficient in *Nr5a1* become obese due to a lack of activity and decreased energy expenditure rather than increased food intake (Flak et al., 2020; Kim et al., 2011; Majdic et al., 2002). Also, *Nr5a1*-expressing cells of the VMH are a heterogeneous group. Selective knockout of leptin receptor (Dhillon et al., 2006) or cannabinoid receptor 1 (Cardinal et al., 2014) from *Nr5a1*-expressing cells leads to high-fat diet-induced dysregulation of energy homeostasis and increased body weight. In contrast, insulin receptors (Klöckener et al., 2011), *Sirt1* (Ramadori et al., 2011), or the *Foxo1* transcription factor (Kim et al., 2012) deficient *Nr5a1*-expressing cells alleviate weight gain related to diet. Therefore, it is difficult to discern the cumulative or combined effects of this heterogeneous group of cells when targeting VMH *Nr5a1*-expressing cells to elucidate the mechanisms of satiety and energy homeostasis.

Cholecystokinin (CCK) is a pleiotropic neuropeptide, a gut hormone, and a well-known peripheral satiety signal synthesized, among others, in the intestinal tract, extraintestinal endocrine glands, and peripheral nervous system (Ahn et al., 2022; Cawthon and de La Serre, 2021; Gibbs et al., 1973; Steinert et al., 2017). Exogenous CCK reduces meal size and duration. However, a compensatory increase in meal frequency allows for regulating total food intake or body weight (Cawthon and de La Serre, 2021; Overduin et al., 2014; West et al., 1984). Peripheral injections of exogenous CCK-8 act on the cholecystokinin A receptors (CCKAR) found mainly on the vagal afferents of the stomach and liver (Reidelberger et al., 2003) but also in the hypothalamus, basal ganglia, thalamus and other areas of the CNS (Sjöstedt et al., 2020). They transmit signals through the vagal nerve to the brainstem’s satiety centers, such as the nucleus tractus solitarius (NTS), resulting in a brief reduction in food intake (Bray, 2000). Antagonists for the CCKAR have been shown to modulate food consumption in a dose-dependent manner across various contexts and species, predominantly by augmenting the size of individual meals (Argueta et al., 2019; Moran et al., 1992, 1993; Reidelberger et al., 2003), while a CCKAR agonist has been shown to lower food intake and body weight of Göttingen Minipigs (Christoffersen et al., 2020).

In the central nervous system (CNS), CCK is highly expressed in the hypothalamus, hippocampus, cerebral cortex, and spinal cord. However, research on its central role in regulating energy expenditure and food intake is still limited. Small sulphated CCK-8 is the predominant form in the CNS (Rehfeld, 1978; Rehfeld and Hansen, 1986). The main CCK receptor in the brain is CCKBR, which is moderately expressed in the dorsomedial and central VMH (Flak et al., 2020). CCKAR is more commonly expressed in the ventrolateral VMH (vlVMH) (Xu et al., 2012) in females controlling their sexual behaviors. It is well known that CNS-derived CCK acts centrally to modulate energy balance by modulating the activity of hypothalamic neuronal populations. CCK-8 injections into different hypothalamic nuclei suppress feeding in rats (Blevins et al., 2000), while CCKAR-deficient rats become hyperphagic (Bi et al., 2001, 2004). Earlier studies on cats and owl monkeys have shown CCK release postprandially from the hypothalamus (Schick et al., 1986, 1987, 1989). Similar findings were reported in lean Zucker rats, where CCK was released postprandially from the PVH, though this response was diminished in obese rats (De Fanti et al., 1998).

Furthermore, injecting a CCK antibody directly into the cerebral ventricles stimulated food intake in sheep (Della-Fera et al., 1981). Following central injections of CCK receptor antagonists, a similar increase in food intake was observed in rats (Corp et al., 1997; Dorré and Smith, 1998; Ebenezer, 2002). Reidelberger and colleagues extended this research by treating vagotomised rats with devazepide and A-70104, both CCKAR antagonists that either penetrate or do not penetrate the blood–brain barrier, respectively. While both compounds stimulated food intake in non-vagotomised control rats, only devazepide increased food intake in rats with subdiaphragmatic vagotomies, underscoring the role of central CCK action in satiety regulation (Reidelberger et al., 2004).

While CCK-expressing neurons are widely distributed throughout the hypothalamus (Geibel et al., 2014; Schiffmann and Vanderhaeghen, 1991), the source of endogenous CCK that acts on distinct neuronal components has not been clarified. Current research does not explain the function of CCK neurons in specific nuclei of the hypothalamus and their likely roles in network dynamics related to energy balance and food intake.

Here, we have untangled the role of CCK-expressing neurons in the hypothalamic circuits responsible for energy homeostasis, specifically their importance in feeding circuits and regulation of body weight. The VMH contains an abundance of CCK-expressing neurons, and this nucleus has long been recognized to affect the regulation of food intake and body weight balance as well as glucose sensing and homeostasis. We have, therefore, focused our studies on the role of CCK-expressing neurons within the VMH (CCK^VMH^).

We used a chemogenetic method to control the activity of CCK-expressing neurons in the VMH. The spatiotemporal control was achieved by using the BAC-CCK-Cre mouse line we had previously established (Geibel et al., 2014) and by intracranially injecting Cre-dependent viral particles expressing Gi-coupled Designer Receptors Exclusively Activated by Designer Drugs (DREADDs) to silence their activity specifically. This approach allowed us to assess their impact on food intake. We used a similar approach to selectively ablate CCK^VMH^ neurons. This was achieved by intracranial injection of a Cre-dependent viral vector that expresses Diphtheria toxin A subunit (DTA) in the VMH of the BAC-CCK-Cre mouse line, leading to the specific ablation of CCK^VMH^ neurons. The latter has helped address the long-term consequences of CCK^VMH^ neuronal loss within the hypothalamus, such as body weight regulation and glucose homeostasis.

## 2 Materials and methods

### 2.1 Animals

All animal procedures conformed to the United Kingdom legislation (Scientific Procedures Act 1986) and the University of Oxford ethical review committee policy, with a final ethical review by the Animals in Science Regulation Unit (ASRU) of the United Kingdom Home Office. Mice were maintained under standard laboratory conditions with *ad libitum* access to food and water unless otherwise specified. Experimental groups consisted of mice of the same strain, sex, and similar age and body weight. Mouse lines used in this study are the BAC transgenic line (BAC-CCK-Cre) expressing Cre recombinase under the CCK promoter previously generated in our laboratory (Geibel et al., 2014) and the Gt(ROSA)26Sor^tm9(CAG-tdTomato)Hze^ reporter line (Madisen et al., 2010), here called Ai9 reporter line. Crossing the BAC-CCK-Cre strain to the Ai9 reporter line generated the BAC-CCK-Cre; Ai9 (here called Ai9-CCK).

### 2.2 Transcriptomic database analysis

HypoMap single-cell gene expression atlas of the murine hypothalamus (Steuernagel et al., 2022) was used to characterize the *Cck*-expressing cells within the VMH of 2 months old mice. UMAP expression of selected genes relevant to energy homeostasis was highlighted specifically in the VMH region with log-normalized expression values scaled to the maximum of each gene. Only positive expression values were shown on the hypothalamus background. A co-expression analysis was performed to show cells expressing *Cck* and other relevant genes on one UMAP, highlighting the cells co-expressing *Cck* and the other gene of interest. Gene expression of relevant genes in the entire hypothalamus was analyzed using the CELLxGENE Discover platform. Specifically, the study by Steuernagel et al., 2022 was selected to analyse hypothalamic neurons. The analysis was fully performed using the toggles of the online CELLxGENE Discover platform and HypoMap atlas (Abdulla et al., 2023; Megill et al., 2021; Steuernagel et al., 2022).

### 2.3 Histology and immunohistochemistry

Mice were perfused as previously described (Gage et al., 2012). Briefly, mice were terminally anesthetised with 50 μL of sodium pentobarbital 200 mg/mL concentration. Brains were prefixed by cardiac perfusion with 20 mL ice-cold 4% paraformaldehyde (PFA) following blood clearance with 0.1 M phosphate buffer saline (PBS, pH 7.4). Brains were post-fixed in 4% PFA/0.1 M PBS for 3 h, then cryoprotected in 10, 20, and 30% sucrose/0.1 M PBS overnight. Brains were embedded in optimal cutting temperature (OCT) compound, snap-frozen in isopentane cooled by dry ice, and stored at −80°C. Sectioning was performed on a Leica cryostat, generating 20 μm thick sections. Sections were washed in PBS and stored at 4°C in PBS with 0.01% sodium azide.

#### 2.3.1 Immunofluorescence and cell quantification

Free-floating sections 20 μm thick were transferred to 24-well plates and processed at room temperature. Sections from BAC-CCK-Cre intracranially injected with AAV carrying mCherry, were blocked with 5% fish gelatin and 0.5% Triton X-100 in PBS for 1 h, then incubated with rabbit anti-dsRed antibody (1:1000, Clontech, 632496) overnight. After washing, sections were incubated with Alexa-555 goat anti-rabbit antibody (1:1000, Invitrogen, 21428) for 2–3 h. DAPI counterstaining (1:10000, 5 mg/mL stock) was performed for 5 min. Sections from Ai9-CCK mouse brains were only stained with DAPI. Other antibodies used were FOXO1 [1:200, New England Biolabs, (C29H4) Rabbit mAb #2880]; anti-POMC precursor (1∶4000, Phoenix Pharmaceuticals, H-029-30); CCK-pro-Rb-Af350 (a gift from Prof. Masayoshi Watanabe, Frontier Institute Co. Ltd); and secondary antibodies, Alexa fluor 488 goat anti-rabbit (1:1000, Invitrogen, A-11008). All sections were mounted on slides, dried overnight, coverslipped with Vectashield (Vector Labs), and stored at 4°C. Imaging was performed using a Leica DM6000B microscope (camera: DFC365FX; objectives: HCX PL APO10x/0.40 CS, HC PL 20x/0.75 CS2 and HCX PL APO 40x/0.85 CORR CS; Leica Microsystems, Wetzlar, Germany). Leica microscope Fluorescence filter cube L5 ET 504166, k; Fluorescence Filter TX2 ET 11504170, k; Leica Microsystems Filter cube LED 405 nm.

VMH Ai9-CCK (tdTomato-expressing) and mCherry-expressing cell numbers were quantified from three mice using three non-consecutive sections spaced 100 μm per mouse. Cell counts were performed manually in NIH ImageJ, with the experimenter blind to the condition.

#### 2.3.2 DAB (3′3-diaminobenzidine) staining

DAB staining was performed on 20 μm thick floating sections. All steps were performed at RT on a rocking platform unless otherwise stated. Sections were washed three times with PBS for 15 min. Endogenous peroxidase activity was quenched by incubating for 20 min in 3% H_2_O_2_. Three 10-min washes in PBS were performed before blocking with 5% fish gelatine and 0.5% Triton-X 100 in PBS for 1 h. Sections were incubated with primary antibody rabbit anti-dsRed antibody (1:1000, Clontech, 632496) diluted in blocking buffer overnight at room temperature. Afterwards, sections were washed thrice with PBS 0.1% Triton for 15 min. The secondary antibody biotinylated donkey anti-rabbit (Jackson, cat. no. 703–065-155) was applied at a dilution of 1:200 in blocking buffer and incubated for 2 h at room temperature. Sections were then washed three times for 15 min with PBST. The peroxidase Vectastain ABC system from (Vector Biolabs, cat. no. PK-6100) was used to develop the staining. The sections were then mounted on gelatine-coated slides and dried overnight. Sections were dehydrated for 1 min in 90% and then 100% ethanol, incubated for 10 min in xylene and then coverslipped with Vectamount. Slides were air-dried for at least a day and then imaged as above.

### 2.4 Electrophysiology recording

#### 2.4.1 Brain slice preparation

Mice were anesthetised with isoflurane and then decapitated. Brains were removed, and coronal 350–400 μm slices were cut using a vibrating microtome (Microm HM650V). Slices were prepared in artificial cerebrospinal fluid (aCSF) containing (in mM): 65 Sucrose, 85 NaCl, 2.5 KCl, 1.25 NaH_2_PO_4_, 7 MgCl_2_, 0.5 CaCl_2_, 25 NaHCO_3_ and 10 glucose pH 7.2–7.4, bubbled with carbogen gas (95% O_2,_ 5% CO_2_). Slices were immediately transferred to a storage chamber containing aCSF (in mM): 130 NaCl, 3.5 KCl, 1.2 NaH_2_PO_4_, 2 MgCl_2_, 2 CaCl_2_, 24 NaHCO_3_ and 10 glucose pH 7.2–7.4, at 32°C and bubbled with carbogen gas until used for recording. Slices were transferred to a recording chamber and continuously superfused with aCSF bubbled with carbogen gas with the same composition as the storage solution (32°C and perfusion speed of 2 mL/min). Whole-cell current-clamp recordings were performed using glass pipettes, pulled from standard wall borosilicate glass capillaries and containing (in mM): 110 potassium gluconate, 40 HEPES, 2 ATP-Mg, 0.3 Na-GTP, 4 NaCl and 4 mg/mL biocytin (pH 7.2–7.3; osmolarity, 290–300 mOsmol/l). Recordings were made using Multiclamp 700B amplifiers and acquired using WinWCP software (University of Strathclyde, United Kingdom).

#### 2.4.2 Slice recording protocols

Recordings of resting membrane potential were performed in whole-cell current-clamp mode. After a 5–10 min recording of baseline resting membrane potential, the hM4Di agonist clozapine-*N*-oxide (CNO; 10–20 μM) was superfused for 15–20 min while continuously measuring the resting membrane potential. Subsequently, CNO was washed out for more than 10 min while continuously measuring the resting membrane potential - we did not observe a significant reversal of hyperpolarisation upon washout. Hyperpolarising and depolarising current steps were used to assess further the intrinsic properties of the recorded neurons, including input resistance and action potential frequency.

#### 2.4.3 Analysis of slice recordings

Data were analyzed offline using custom-written procedures in Igor Pro (Wavemetrics). Resting membrane potential measurements were taken from the last 10 s of the baseline recordings and the last 10 s of the CNO superfusion recordings. The input resistance was calculated from the observed membrane potential change after hyperpolarizing the membrane potential with a set current injection. The membrane time constant was taken as the time it takes for a change in potential to reach 63% of its final value. The action potential amplitude was taken from the peak amplitude of the individual action potentials relative to the average steady-state membrane depolarization during positive current injection. Action potential duration was taken as the duration between the upward and downward stroke of the action potential at 25% of the peak amplitude.

### 2.5 Intracranial injections

#### 2.5.1 Adeno-associated viral particles and bregma

AAV vectors used in this study: AAV9-CAG-FLEX-DTA-IRES-mCherry (Virovek, Lot 15–335) (here called AAV-DIO-DTA) expressing Diphtheria toxin A subunit (DTA) and mCherry fluorescent protein in a Cre-dependent manner under the CAG promoter. A control vector, AAV9-CAG-mCherry (Virovek, Lot#11–148). And AAV1-CAG-FLEX-eGFP-WPRE-bGH (Penn Vector Core) (here called AAV1-DIO-eGFP). We also made use of AAV2/5-hSyn-DIO-hM4Di-mCherry (here called AAV-hM4Di) (200 nL at 5.1 × 10^12^ GC/ml; University of North Carolina vector core) expressing the inhibitory G protein-coupled receptor hM4Di Designer Receptors Exclusively Activated by Designer Drugs (DREADDs) in a Cre-dependent manner. Expression was driven by the hSyn promoter, and the hM4Di was fused to the mCherry protein at the C-terminus. Activation of hM4Di in neurons inhibits neuronal activity by triggering hyperpolarisation through Gβ/*γ*-mediated activation of G-protein inwardly rectifying potassium channels and suppressing neurotransmitter release at the presynaptic level (Armbruster et al., 2007). And the AAV2.EF1a.DIO.eYFP.WPRE.hGH (Addgene27056) (here called AAV2-DIO-YFP), with an EF1a-driven, Cre-dependent EYFP expression. Finally, FluoSpheres™ green-fluorescent 0.04 μm diameter, Invitrogen, cat no: F-8795, were used to establish VMH intracranial injection coordinates.

#### 2.5.2 Stereotactic surgery

Mice were anesthetised with 2–3% vaporised isoflurane and placed in a stereotactic frame. An analgesic cocktail was administered, and the skull was exposed. Coordinates were adjusted based on the Allen Mouse Brain Atlas. For targeting the whole VMH nucleus, the coordinates were adjusted to AP 1.5 mm, ML ±0.6 mm and DV -6.5 mm. Burr holes were drilled, and viral particles were injected using a Hamilton syringe (34-gauge, 30 mm length) at a rate of 125 nL/min. The needle was left in place for 10 min post-injection to prevent reflux. Incisions were sutured, and mice were allowed to recover in a pre-warmed incubator before returning to their home cages. Post-operative care included Metacam administration as needed. The animals were allowed to recover for 3–4 weeks before any experiments, ensuring sufficient expression of AAVs in the brain.

### 2.6 Food intake measurements and analysis

#### 2.6.1 Metabolic cages

After surgery (3 to 4 weeks), the mice were transferred into the LabMaster metabolic cages (TSE Systems). These cages were fitted with calibrated sensors capable of measuring food and water intake by calculating the average weight changes of the food hopper in ten-second intervals, with a sensitivity of 0.01 g. Food and water were provided *ad libitum*. Mice were fed the standard chow diet, Teklad Rodent Diet (60% calories from carbohydrates, 23% from protein and 17% from fat, 3.3 Kcal/g digestible energy). Two days before the experiment, mice were habituated to the new water bottle nozzle, which releases water upon touch. Mice were singly housed in the metabolic cages and allowed at least a week to habituate to the new environment before recording any food sensor measurements. Food intake was measured continuously. The cages were inspected daily for any dropped pellets, and any small pellets were removed from the food hopper to avoid drops. A meal event (meal bout) was defined as the total of all sensor measurements taken less than 15 min apart (minimum intermeal interval). The minimum meal size was set at 0.02 g, a commonly used method for defining meals in mice and rats (Atalayer and Rowland, 2011; Bake et al., 2014; Farley et al., 2003). Meal metrics included size, duration in minutes, and intermeal interval, averaged across the experimental period, daily (average of days), or in specific hours (i.e., 6 h post hM4Di activation).

#### 2.6.2 Pharmacogenetic hM4Di inhibition

Mice underwent stereotactic surgery as described above and were injected bilaterally with AAV2/5-hSyn-DIO-hM4Di-mCherry in the VMH region. After 3–4 weeks of recovery, the mice were habituated to handling and intraperitoneal (IP) injections. IP injections of sterile saline with 10% DMSO (dimethylsulfoxide) were administered at 10 a.m. and 6 p.m. daily for 10 days to habituate the animals to the injections. Food intake data was collected during the habituation period and was monitored and analyzed as described. Following habituation, animals received increasing doses of CNO (Tocris Biosciences, cat. no. 4936) via IP injections twice daily. Appropriate non-AAV-hM4Di injected littermate controls were used to control the CNO reverse-metabolisation to clozapine, which produces clozapine-like interoceptive stimulus effects in mice (Manvich et al., 2018). The doses were 0.75, 1.5, 2, and 3 mg/kg body weight, each administered for 3 days. Food intake measurements and meal pattern analysis were performed as described above (2.6.1 section). Before each injection, cage sensors were paused for 1 min, and animals were handled minimally to ensure consistent treatment.

### 2.7 Diphtheria toxin deletion of CCK^VMH^ neurons

Ai9-CCK and Ai9-Control (not expressing Cre) mice were injected bilaterally with AAV9-CAG-FLEX-DTA-IRES-mCherry in the VMH region, as described in section 2.5.2 above. Their body mass was monitored for 3 months post-surgery. A month after the surgery, the mice were singly caged in metabolic cages, and their food intake was measured starting with a 10-day habituation period and for five experimental days, after which the measurements were averaged.

### 2.8 Glucose tolerance test

Female Ai9-CCK and Ai9-Control mice were injected with AAV9-CAG-FLEX-DTA-IRES-mCherry in the VMH nucleus and allowed to recover for 3 months. They were fasted overnight (16 h) before the glucose tolerance test. Blood glucose levels were measured using a hand-held glucose meter (One Touch Verio). Following a baseline glucose reading, mice received an IP injection of D-glucose (200 g/L) at 2 g/kg body weight. Blood glucose levels were measured at 30- and 180 min post-injection. Mice were sacrificed by transcardial perfusion, and their brains were harvested.

### 2.9 Statistical analysis

Experiments and analyses were conducted blind to genotype with at least three animals per genotype. Unless stated otherwise, values were presented as mean ± standard error of the mean (s.e.m). Statistical significance was assessed using paired/unpaired two-tailed Student’s t-test, one- or two-way ANOVA with appropriate post-hoc tests, either Dunnett’s or Sidak’s *post hoc* test or Fisher’s LSD test, as appropriate for each data set and indicated in the figure legends. Significance was defined as *p* < 0.05. Analyses were performed using GraphPad Software (La Jolla, CA, United States).

## 3 Results

### 3.1 Characterization of the CCK-expressing cells in the ventromedial hypothalamus

**Figure 1:**
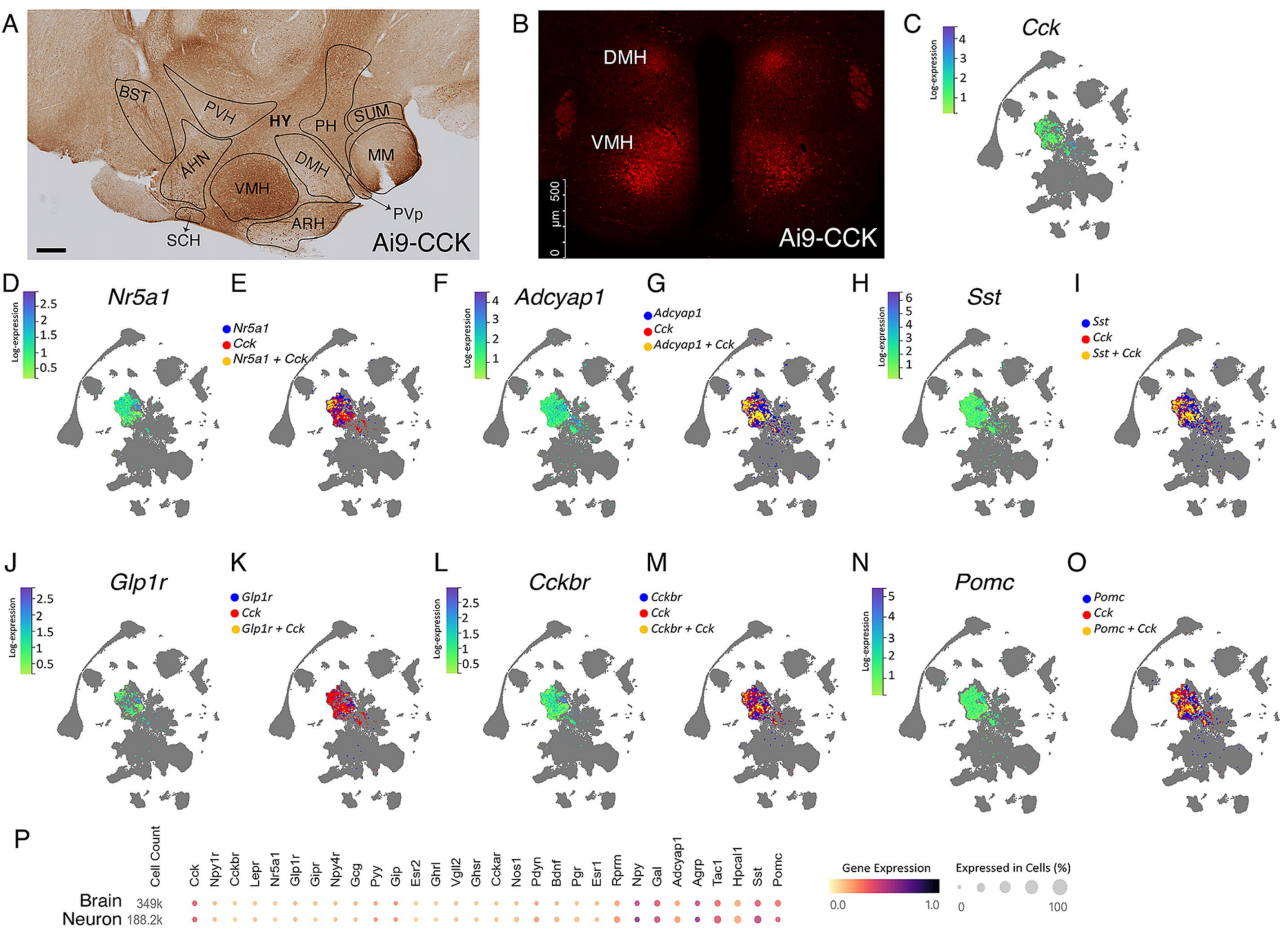
Single-cell transcriptomic analysis reveals heterogeneity of the CCK^VMH^-expressing neurons. (A,B) Representative immunohistochemical and IF images of CCK + tdTomato expressing cells in the hypothalamic regions from the Ai9-CCK brain sections. (A) Tile image of DAB-stained sagittal section showing, in particular, the hypothalamic region highlighting the different nuclei, such as ventromedial hypothalamus (VMH), dorsomedial nucleus (DMH), anterior hypothalamic nucleus (AHN), and (B) IF image from a coronal section showing endogenously expressed td-Tomato in CCK-expressing (CCK + tdTomato) cells around the paraventricular hypothalamic nucleus (PVH) in the DMH and the VMH. (C,D,F,H,J,L,N) HypoMap (Steuernagel et al., 2022) UMAP expression of selected genes relevant to energy homeostasis in the VMH, showing a specific selection of only positive expression values in the VMH region of 2-months old mice. The color sidebar corresponds to log-normalized expression values scaled to the maximum of each gene, with the rest of the hypothalamus in gray. (E,G,I,K,M,O) HypoMap (Steuernagel et al., 2022) UMAP of cells expressing selected genes relevant to energy homeostasis in the VMH, with marked cells expressing *Cck* in red, the other selected genes in blue and cells co-expressing the relevant gene with *Cck* in yellow. Only positive expression values are shown. (P) Relevant gene expression from a study utilizing the mouse hypothalamus (Steuernagel et al., 2022), with specific data for the hypothalamic neurons highlighted, prepared using the CELLxGENE Discover platform (Abdulla et al., 2023). HY, hypothalamus, PH, posterior hypothalamic nucleus, SUM, supra mammillary nucleus, MM, medial mammillary nucleus, PVp, periventricular hypothalamic nucleus (posterior part), BST, Bed nuclei of the stria terminalis. Scale bars, panel A, 300 μm; panel B, 500 μm.

To specifically target the CCK^VMH^ neurons, we used the BAC-CCK-Cre mouse line, previously generated and characterized in our laboratory (Geibel et al., 2014). We first confirmed this line’s recombination pattern by crossing the BAC-CCK-Cre strain with the Ai9 reporter line (Madisen et al., 2010), leading to tdTomato fluorescence signal upon Cre-recombination (Ai9-CCK). Focusing in particular on the hypothalamic region and the nuclei adjacent to the VMH nucleus, we confirmed our previous findings; CCK-expressing neurons were visualised in both sagittal and coronal sections using immunohistochemical staining in 3,3′-diaminobenzidine (DAB) or direct fluorescent imaging of tdTomato (Figures 1A,B). In particular, we identified CCK + tdTomato expressing neurons in the anterior hypothalamic nucleus (AHN), DMH, and VMH nuclei, with some presence in the ARH and suprachiasmatic nucleus (SCH) and very few in the PVH.

Using the HypoMap single-cell gene expression atlas of the murine hypothalamus (Steuernagel et al., 2022), we demonstrate that at 2 months of age, the majority of CCK^VMH^ cells do not co-express the *Nr5a1* gene, which encodes SF1 (Figure 1E). Only around 10% of CCK^VMH^ cells co-express *Nr5a1*, with a varying degree of expression of both *Cck* and *Nr5a1* (Figures 1C,D). The CCK^VMH^ cells are a heterogeneous group co-expressing various genes involved in energy homeostasis. Approximately 58% of them co-express *Adcyap1* encoding PACAP, which is known to regulate metabolic rate and energy balance and has a high expression in the VMH (Bozadjieva-Kramer et al., 2021; Figures 1F,G). In addition, about 36% of CCK^VMH^ co-express somatostatin (*Sst*), which is important for regulating factors in the satiety system and obesity. This includes both central and peripheral effects, such as increasing food intake by inhibiting the release of endogenous CCK and GLP-1 (Kumar and Singh, 2020; Figures 1H,I). GLP-1 receptor agonists are at the forefront of hormone-based treatments for obesity (Müller et al., 2019). While GLP-1 is not expressed in the hypothalamus, its receptor GLP-1R is co-expressed with approximately 1.5% of CCK^VMH^ cells (Figures 1J,K). The main CCK receptor in the brain, CCKBR (Flak et al., 2020), is expressed in a greater number of cells of the VMH than CCK, but only 12% of those co-express both (Figures 1L,M). Hypothalamic *Pomc-* and *Agrp-*expressing neurons are conventionally considered to function in opposing ways, contributing to anorexigenic and orexigenic signaling, respectively (Quarta et al., 2021). *Pomc* is co-expressed with approximately 25% of *Cck*-expressing cells of the VMH and 23% in the ARH (Figure 1N,O; Supplementary Figures S1A–C). *Foxo1*, a well-known negative leptin signaling regulator (Quarta et al., 2021), is co-expressed with approximately 5% *Cck*-expressing VMH cells (Supplementary Figures S1G,H). Finally, CCK^VMH^ cells also co-express *Bdnf* (34%), *Tac1* (30%), *Esr1* (26%), *Agrp* (16%), *Mc3r* (9.5%), and *Lepr* (8%) among some of the genes related to energy homeostasis, which is paralleled in neurons on the scale of the entire hypothalamus (Figure 1P). We have validated some of these RNAseq results through immunofluorescence colocalisation studies (Supplementary Figures 1D–F,I–K), confirming a faithful BAC-CCK-Cre strain recombination pattern.

### 3.2 Pharmacogenetic silencing of CCK^VMH^ neurons results in increased food intake and alterations in meal patterns

**Figure 2:**
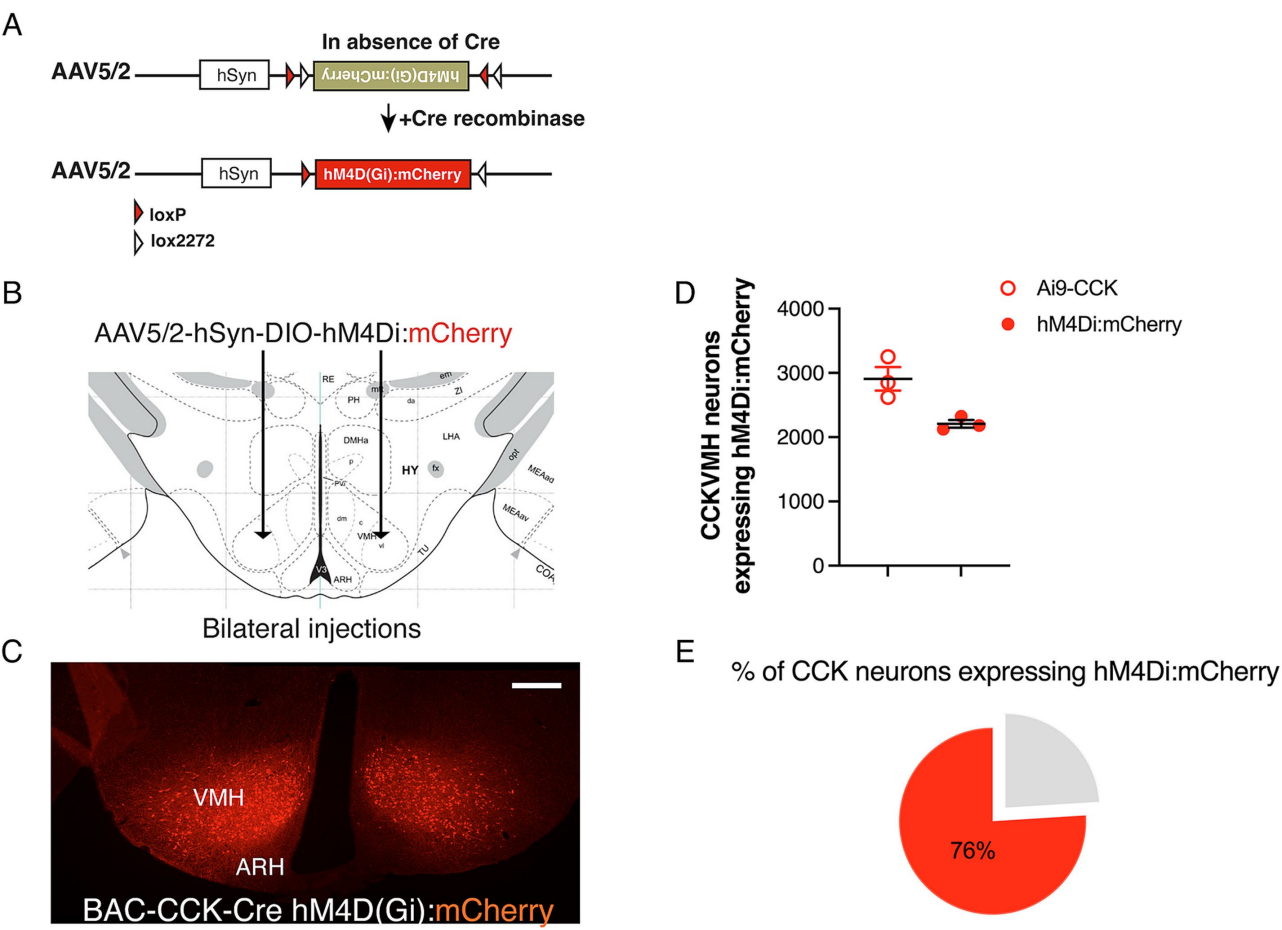
Successful Cre-dependent expression of inhibitory DREADDs in CCK^VMH^ neurons of BAC-CCK-Cre mice. (A) Diagram of the Double-floxed Inverted Orientation (DIO) AAV construct depicting the Cre-dependent recombination/inversion of the hM4D(Gi):mCherry cassette into the sense orientation to allow its expression in Cre-positive cells (Cre + ve). (B) Drawing of the bilateral stereotactic injection of the AAV-DIO-hM4Di(Gi):mCherry in the VMH region. (C) Successful expression of hM4Di(Gi):mCherry in the CCK^VMH^ neurons of BAC-CCK-Cre hypothalamic area 2 weeks after surgery. (D) Quantification of neurons expressing hM4Di compared to the CCK^VMH^ neurons of Ai9-CCK reporter line (see Figure 1B). CCK^VMH^ neurons expressing hM4Di, 2209 ± 60 mCherry-positive neurons out of 2908 ± 184 Ai9-CCK reporter line detected CCK-expressing neurons (*n* = 3 male mice per group). (E) About 76% of CCK^VMH^ neurons expressed hM4Di. Scale bar panel C, 200 μm.

To investigate the short-term role of CCK^VMH^ neurons, we employed the DREADDs pharmacogenetic approach (Roth, 2016). This technique allowed for rapid neuronal silencing following the systemic administration of CNO. Stable expression of the inhibitory hM4Di in CCK^VMH^ neurons was achieved via stereotactic delivery of AAV-hM4Di bilaterally at established VMH coordinates (Supplementary Figure S2). The AAV construct included a DIO cassette for Cre-dependent expression of the hM4D(Gi) receptor and a mCherry fluorescent protein for cell identification. Stereotactic surgery was performed on young adult BAC-CCK-Cre animals by injecting 200 nL at 5.1 × 10^12^ GC/ml of AAV-hM4Di bilaterally (Figures 2A,B). Immunohistochemical analysis confirmed hM4Di expression in CCK^VMH^ neurons of injected mice (hM4Di-CCK^VMH^). This was determined by detecting the endogenous mCherry fluorescence, indicating that the viral spread was limited to the VMH region, as expected (Figure 2C). Quantifying the transduced hM4Di:mCherry-CCK^VMH^ neurons revealed that 76% expressed hM4Di:mCherry (Figures 2D,E).

**Figure 3:**
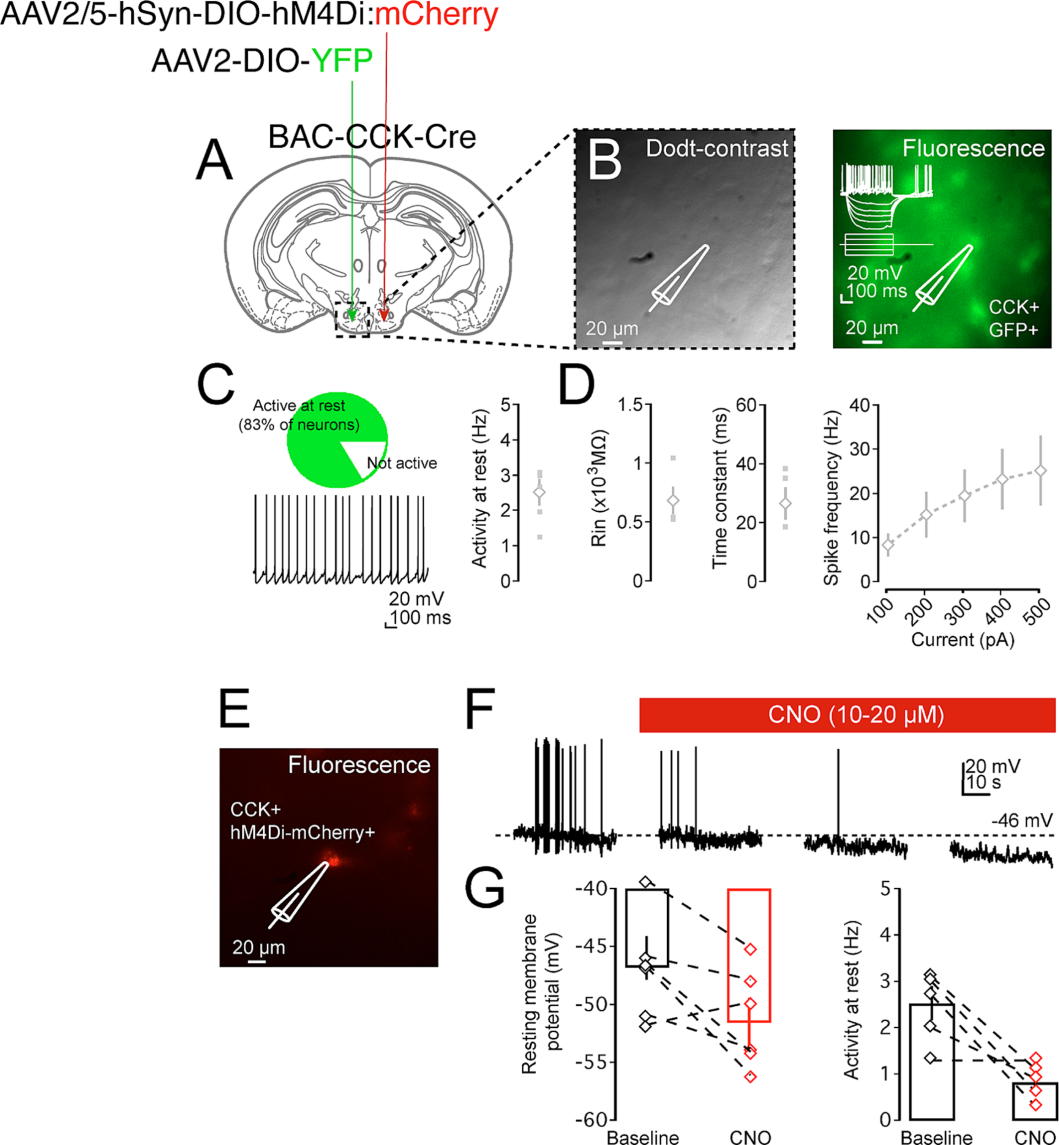
Pharmacogenetic inhibition of CCK^VMH^ neurons in brain slices. (A) A diagram of a coronal brain section showing an experimental design for a BAC-CCK-Cre injected with appropriate viruses on the two sides of the hypothalamus-VMH area. The recording area containing the VMH is indicated in the dashed box. (B) Dodt-contrast image of the VMH hypothalamic region with the placement of recording electrode and neuron during patch-clamp recordings (left). Fluorescent image indicating that the recorded neuron is a CCK+ GFP+ neuron (right). (C) CCK^VMH^ neurons are mostly spontaneously active, and further intrinsic properties suggest they are small, high Rin neurons with a firing rate of between 2 and 20 Hz (D). (E) Fluorescence image indicating a recorded neuron that is hM4Di:mCherry positive. (F) Example of recording trace of spontaneously active CCK^VMH^ neuron. Not only did CNO superfusion lead to a significant hyperpolarisation of the resting membrane action potentials, but this was also concomitant with a reduction in the frequency of spontaneous action potential. (G) Summary plot of the effect of CNO on the resting membrane voltage and firing frequency of the hM4Di-CCK^VMH^ neurons. Hyperpolarisation of recorded neuron upon washing of CNO, baseline: −45.99 ± 1.87 mV and CNO: −51.55 ± 2.10 mV, *p* = 0.016. Reduction in the spontaneous action potential frequency of the recorded neuron upon washing of CNO baseline: 2.53 ± 0.37 Hz and CNO: 0.83 ± 0.21 Hz, *p* = 0.027. *n* = 5. Values are means ± s.e.m. *p* statistic from two-tailed paired student t-test. Scale bar panel B, E 20 μm.

Following the successful expression of hM4Di in the CCK^VMH^ neurons, we first validated the ability of CNO to successfully inhibit the generation of action potentials by recording their firing rate through electrophysiology upon CNO administration. Therefore, we unilaterally injected another group of BAC-CCK-Cre animals (~8 weeks old) with AAV-hM4Di on one hemisphere and a control AAV2-DIO-YFP virus on the other. Three weeks after the injection, acute brain slices containing the hypothalamus were made from these mice and analyzed electrophysiologically. We found that CCK^VMH^ neurons were spontaneously active (83.3%, 5 out of 6 neurons, Figures 3A–C) with an average firing frequency of 2.53 ± 0.37 Hz. The intrinsic properties of CCK^VMH^ neurons suggest they are excitable cells with a high input resistance (854 MΩ) but are slow to respond to inputs as they exhibit a slow membrane time-constant (26.5 ms) concomitant with their function in the hypothalamus (Figure 3D). The spontaneous firing frequency and response to depolarising current steps would suggest they have an action potential firing range roughly between 2 and 20 Hz. Neurons significantly hyperpolarise upon the superfusion of the chemogenetic actuator CNO, followed by a reduction in the spontaneous action potential frequency (Figures 3E–G). These data show that we achieved sufficient expression of hM4Di, which is capable of silencing CCK^VMH^ neurons upon CNO application.

**Figure 4:**
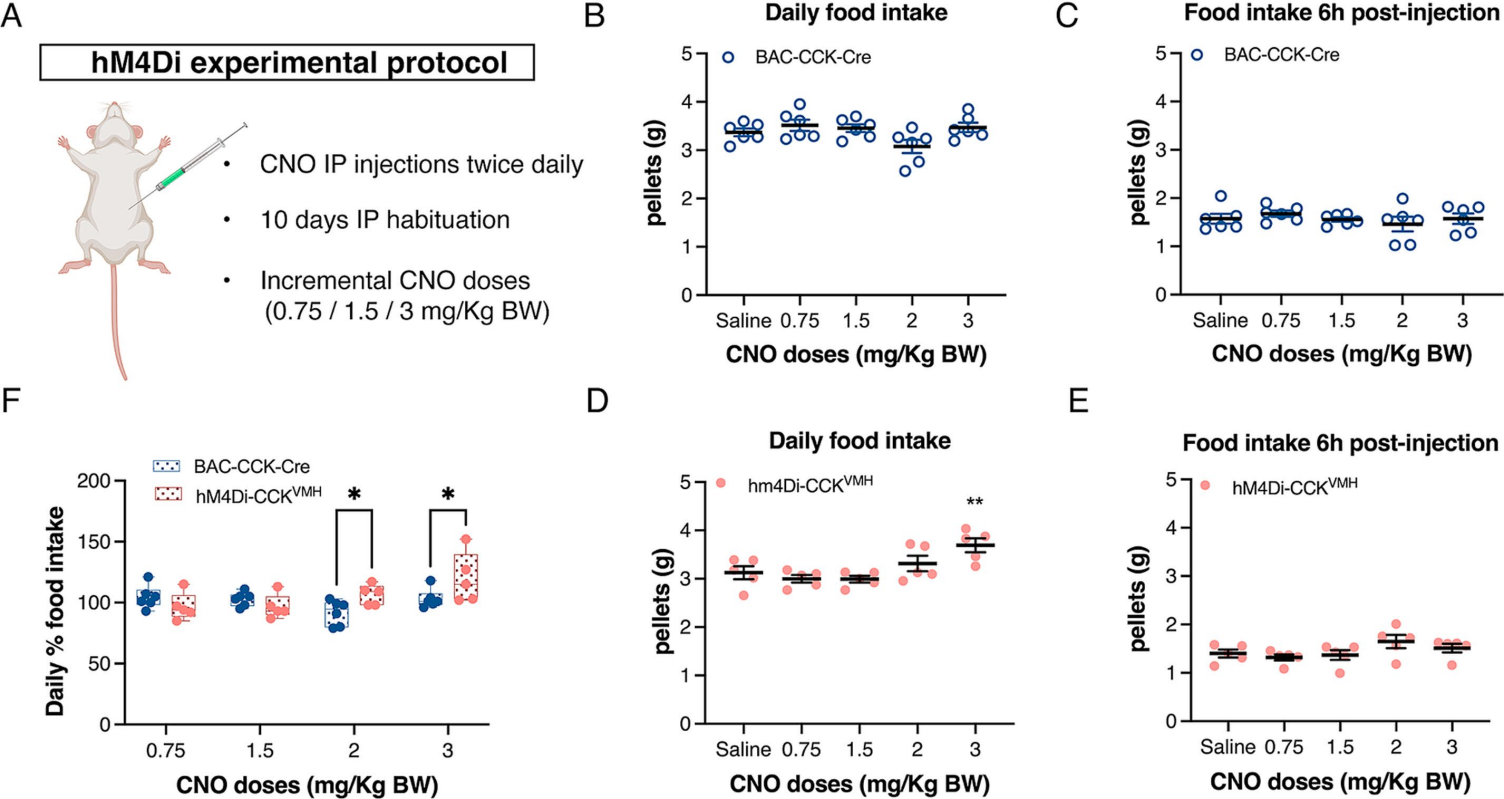
Acute inhibition of CCK^VMH^ neurons causes a gradual increase in total food intake. (A) DREADDs experimental protocol to assess food intake. Animals were singly housed for 2 days in metabolic cages and then habituated for 10 days with saline IP injections twice daily (10 a.m. and 6 p.m.). They were then injected with increasing doses of CNO (0.75/1.5/2/3 mg/kg BW). Each dose was administered for 3 days. (B,C) The plots display the daily food intake of control BAC-CCK-Cre non-injected mice and their food intake 6 h post-injection. At no point was there a significant difference from the saline baseline. (D) Plot showing the daily food intake post-injection for hM4Di-CCK^VMH^ mice. IP 3 mg/kg of BW CNO significantly increased the daily food intake of chow pellets from 3.125 ± 0.135 g to 3.693 ± 0.143 g compared to the saline baseline, *p* = 0.002. (E) Cumulative food intake 6 h post-injection with saline/CNO of hM4Di-CCK^VMH^ animals. (F) Percentage change of daily total food intake of hM4Di-CCK^VMH^ animals compared to the control BAC-CCK-Cre group. Each group was normalized to their own IP saline baseline. Significant increases in hM4Di-CCK^VMH^ mice compared to controls at 2 and 3 mg/kg BW doses (2 mg: 92 ± 4% vs. 106 ± 4%, *p* = 0.032; 3 mg: 103 ± 3% vs. 120 ± 9%, *p* = 0.016). Values are means ± s.e.m. (*n* = 5–6 mice per group, males). *p* statistic (B–E) from repeated measures one-way analysis of variance followed by Dunnett’s *post hoc* test; (F) from two-way analysis of variance followed by Fisher’s LSD test. Image in A was created with BioRender.com.

Given the variability in CNO doses reported in the literature, we titrated the dose range for *in vivo* DREADDs applications. Three to 4 weeks post-intracranial injection, the mice were individually housed in metabolic cages for food intake analysis. Following our established 10-day habituation to saline IP injections (Supplementary Figure S3), animals received incremental CNO doses [0.75, 1.5, 2, and 3 mg/kg body weight (BW)] twice daily, each dose for three consecutive days. Food intake was recorded in hM4Di-CCK^VMH^ and control mice (Figure 4A). As hM4Di activation in neurons typically persists for 6–8 h (Roth, 2016), the CNO temporal effect was examined at 6 h and 24 h post-CNO injections. In control mice, CNO administration did not affect food intake at any concentration within 6 h post-injection or daily (Figures 4B,C).

In contrast, hM4Di-CCK^VMH^ animals, upon activation of hM4Di with 3 mg/kg of BW CNO, significantly increased daily food intake of chow pellets (Figure 4D) compared to saline baseline. Lower doses showed no significant effect (Figure 4D). Food intake analysis as a percentage change relative to baseline saline injections revealed significant increases at 2 and 3 mg/kg BW CNO doses in hM4Di-CCK^VMH^ mice compared to controls (Figure 4F). However, hM4Di-CCK^VMH^ mice showed no significant change in cumulative food intake 6 h post-CNO injection (Figure 4E). Despite the significant increase in daily food intake with 3 mg/kg BW CNO, immediate consumption remained unchanged within the first 6 h post-injection, suggesting a delayed effect. This interesting discovery sparked further questions about regulating food intake in hM4Di-CCK^VMH^ mice.

**Figure 5:**
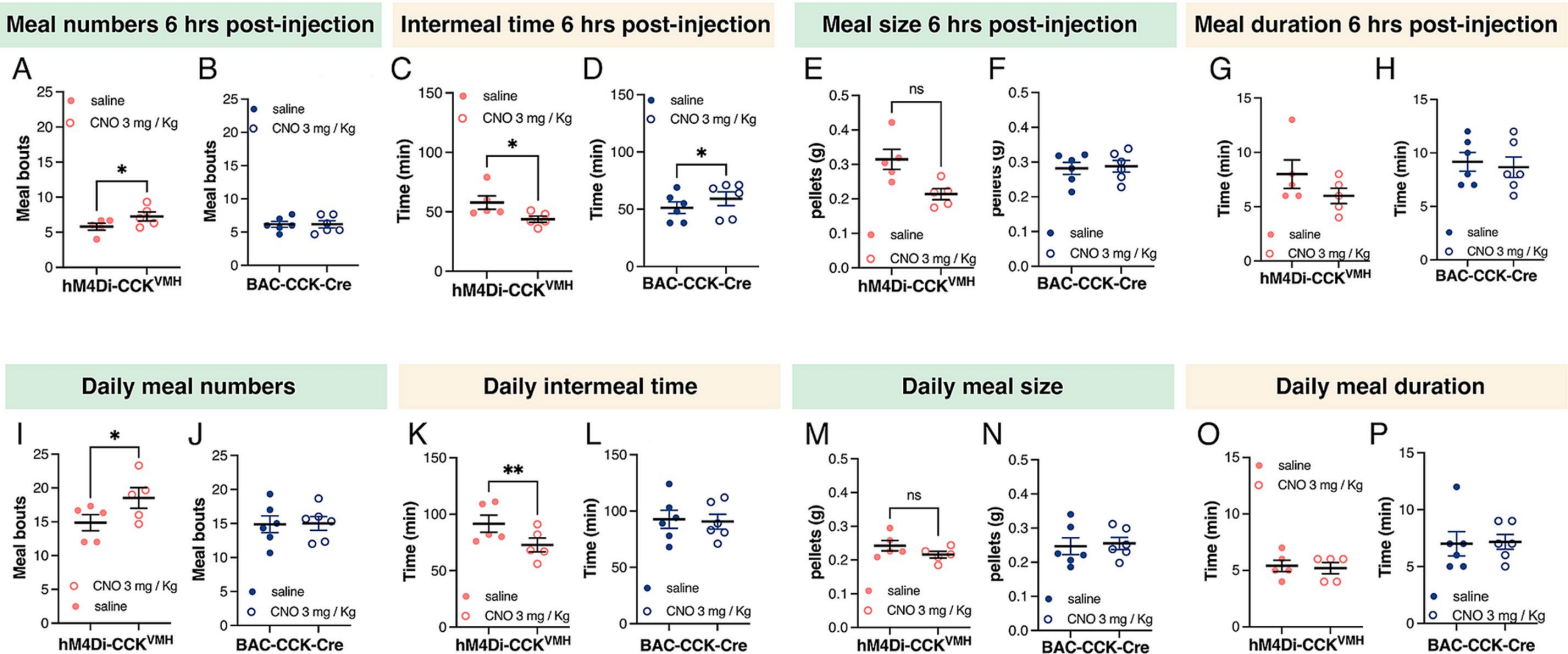
Silencing CCK^VMH^ neurons alters meal patterns. (A–D) Six hours post-CNO administration (3 mg/kg BW) revealed increased meal frequency in hM4Di-CCK^VMH^ animals, from 5.802 ± 0.4904 to 7.266 ± 0.6439 meals, *p* = 0.048 (A) and decreased intermeal time, from 57.80 ± 5.652 min to 43.80 ± 2.615 min, *p* = 0.035 (C). The control group showed no changes in meal frequency (B) and an increased intermeal time from 51.33 ± 5.057 to 59.17 ± 5.997 min, *p* = 0.035 (D). (E–H) Meal size and duration showed no difference in either group. (I–L) Meal pattern alterations persisted throughout the day under CNO administration. hM4Di-CCK^VMH^ animals showed further increased meal frequency from 14.87 ± 1.181 to 18.53 ± 1.514 meals *p* = 0.014 (I) and decreased intermeal time from 91.60 ± 7.580 to 72.80 ± 6.143 min, *p* = 0.006 (K), while control animals exhibited no changes (J,L). (M–P) Daily meal size and duration remained unchanged in both groups. Values are means ± s.e.m. (*n* = 5–6 mice per group, males). *p* statistic from two-tailed paired student *t*-test.

Therefore, we analyzed a meal pattern 6 h post-CNO administration (3 mg/kg BW). The results showed a significant increase in meal frequency in hM4Di-CCK^VMH^ animals and a significant decrease in intermeal interval (Figures 5A,C). Control mice showed no change in meal frequency and an increased intermeal time (Figures 5B,D). Meal size, duration, and consumption rate did not show statistically significant changes in either group (Figures 5E–H). Meal pattern changes persisted throughout the day during CNO administration. hM4Di-CCK^VMH^ animals exhibited significantly increased meal frequency and decreased intermeal time, while control animals exhibited no changes (Figures 5I–L). Daily meal size and duration remained unchanged (Figures 5M–P).

In summary, silencing CCK^VMH^ neurons via CNO-activated DREADDs significantly increased daily food intake and meal frequency, with no immediate effect on consumption within 6 h post-injection. These findings suggest that CCK^VMH^ neurons are critical in regulating meal patterns, contributing to increased cumulative food intake.

### 3.3 Ablation of CCK^VMH^ neurons by AAV-mediated Cre-dependent expression of diphtheria toxin A subunit leads to hyperphagia and obesity

**Figure 6:**
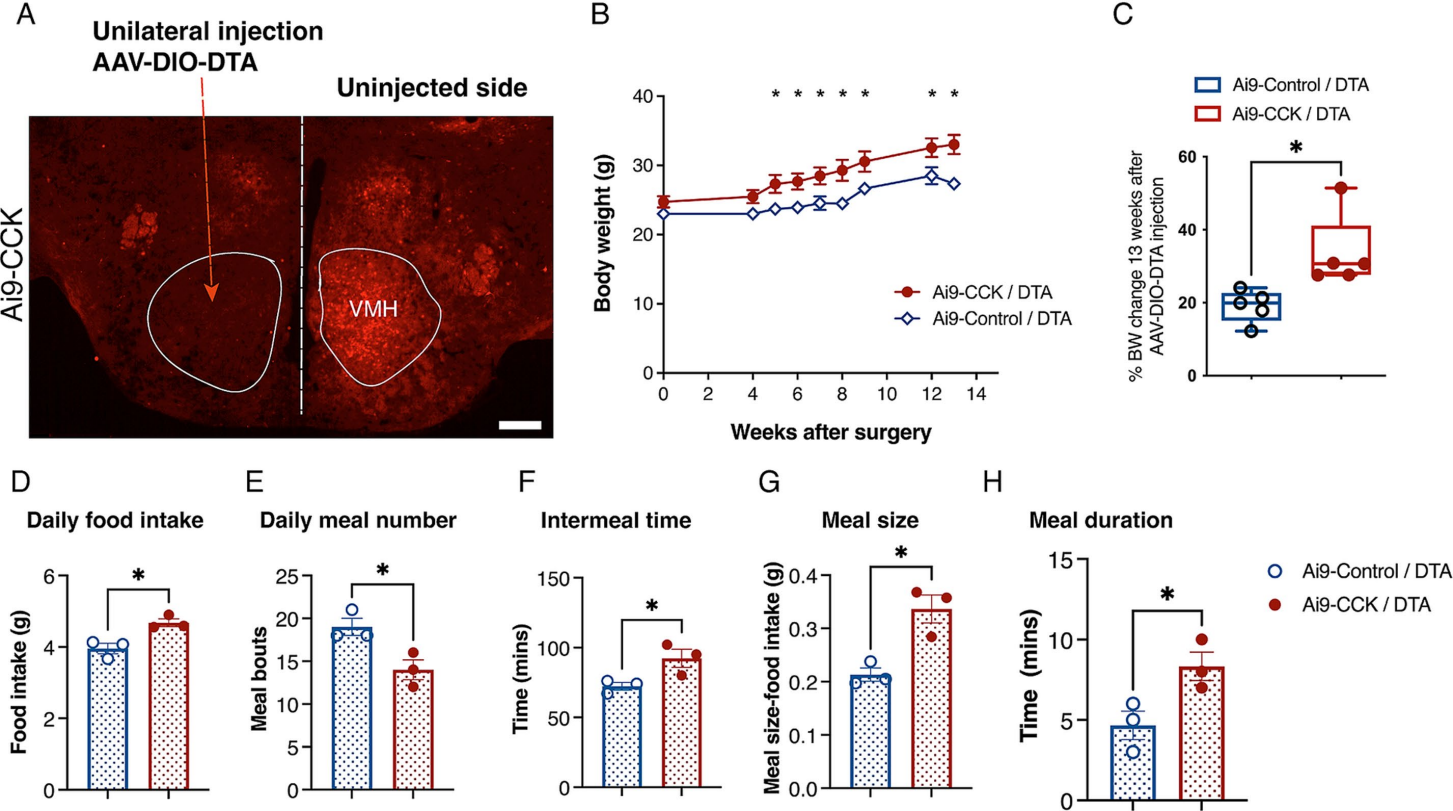
DTA-mediated ablation of CCK^VMH^ neurons in Ai9-CCK mice leads to prolonged weight gain due to increased food intake and disrupted meal patterns. (A) Coronal brain section from Ai9-CCK/DTA injected unilaterally with AAV-DIO-DTA, showing that the Ai9 fluorescent signal significantly decreased in the injected VMH region compared to the contralateral side. (B) A cohort of female mice (n = 5 per group; Ai9-CCK and Ai9-Control) aged 3–4 months were injected bilaterally with AAV-DIO-DTA. BW analysis was recorded for a total of 3 months after surgery. Ai9-CCK/DTA animals significantly increased their BW compared to the Ai9-Control /DTA group starting 5 weeks after injection (Adj *p* = 0.042), culminating with 13 weeks Adj *p* = 0.00147. (C) Three months after surgery, the percentage of BW in each group significantly changes based on its pre-surgery weight. However, there was a significant difference between Ai9-CCK/DTA and the control group (Ai9-Control/DTA, 19.09 ± 1.98 vs. Ai9-CCK/DTA, 33.6 ± 4.50, *p* = 0.018) attributable to the ablation of CCK^VMH^ neurons. (D-H) Food intake and meal pattern analysis between the two groups reveals that Ai9-CCK/DTA animals increased food intake with longer and bigger meals while decreasing their meal frequency. (D) The food intake of the CCK^VMH^-ablated animals increased compared to the control group, as shown by the average daily intake (3.955 g ± 0.1457 vs. 4.679 g ± 0.1098, *p* = 0.016). (E) Meal bouts (19.00 ± 1.00 vs. 14.00 ± 1.15, *p* = 0.0307). (F) Intermeal time (minutes) (72.33 ± 2.72 vs. 92.33 ± 6.489, *p* = 0.047). (G) Meal size (0.2133 g ± 0.012 vs. 0.3366 g ± 0.026, *p* = 0.0133). (H) Meal duration (minutes) (4.667 ± 0.88 vs. 8.333 ± 0.88, *p* = 0.0424). Values are means ± s.e.m. *p* statistic (B) from multiple student t-tests corrected for multiple comparisons using the Holm-Šídák method and (C–H) from two-tailed unpaired student *t*-test. D-H, *n* = 3 female mice in each group. Scale bar panel A, 200 μm.

To investigate the long-term role of CCK^VMH^ neurons in maintaining energy homeostasis, we sought to perform spatiotemporal ablation of the CCK^VMH^ neurons. Therefore, we again used a pharmacogenetic approach, performing intracranial injections to express the diphtheria toxin A subunit (DTA) via AAV-DIO-mediated expression in the CCK^VMH^ neurons. First, we determined an optimal AAV-DIO-DTA virus titre by titration performed in Ai9-CCK control animals (not expressing Cre, here called Ai9-Control/DTA). Injections with various dilutions identified 2.32 × 10^11^ GC/ml as the most tolerable titre, avoiding non-specific weight gain observed at higher titres. Weight monitoring up to 6 months post-surgery revealed no significant deviations in BW between Ai9-Control/DTA injected animals and non-injected controls (data not shown). Successful ablation of CCK^VMH^ neurons was confirmed by a significant reduction in tdTomato fluorescence in the VMH region 1 month after unilaterally injecting AAV-DIO-DTA in Ai9-CCK brains (Ai9-CCK/DTA) compared to the contralateral side used as control (Figure 6A). We conducted an additional experiment to further confirm AAV-DIO-DTA’s specific deletion of CCK^VMH^ cells without affecting non-transduced surrounding cells. In this experiment, we intracranially injected AAV-DIO-DTA and AAV1-DIO-eGFP unilaterally into Ai9-CCK mice. The AAV1-DIO-eGFP was used to identify the location and extent of the Cre-mediated deletion (Supplementary Figure S4). This experiment confirmed a significant reduction of both CCK + tdTomato and eGFP co-expressing cells in the VMH at the site of injection (Supplementary Figures S4A–C). However, moving 200 μm away from the injection site would result in a partial deletion of tdTomato-eGFP expressing cells, suggesting that those not transduced by AAV-DIO-DTA would remain intact in the VMH (Supplementary Figures S4D–I).

We also performed a similar experiment by injecting the AAV-DIO-DTA viral construct into another brain region that expresses higher levels of CCK, specifically the hippocampal CA1 region. This was done to confirm further that the AAV-DIO-DTA targets only the transduced CCK neurons at the injection site, not the surrounding cells (Supplementary Figure S5).

Finally, using the established viral AAV-DIO-DTA titre, Ai9-CCK animals and Ai9-Control littermates (Cre negative) were intracranially injected in the VMH region (resulting in Ai9-CCK/DTA and Ai9-Control/DTA, respectively). All experimental groups underwent surgery at 3–4 months of age and had a weight ranging from 23 to 25 g. BW was recorded for about 13 weeks post-surgery. Ai9-CCK/DTA animals exhibited a significant increase in BW starting around 5 weeks post-surgery, and their BW continued to increase through the experimental time compared to Ai9-Control/DTA (Figure 6B). At 13 weeks post-surgery, Ai9-CCK/DTA animals showed about a 34% increase in BW, while Ai9-Control/DTA animals showed a 19% increase, indicating about 15% difference attributed to CCK^VMH^ neuron ablation (Figure 6C).

However, to examine the effects of CCK-expressing neuron ablation on food intake and meal patterns, new cohorts of Ai9-CCK/DTA and Ai9-Control/DTA animals were singly housed in metabolic cages one-month post-surgery. After a 10-day habituation period to the cages, food intake was recorded in real-time for 5 days and averaged. Ai9-CCK/DTA animals significantly increased daily food intake compared to injected Ai9-Control/DTA animals (Figure 6D). Meanwhile, meal pattern analysis revealed a significant decrease in meal frequency, with the number of daily meals decreasing from 18 to 14 and intermeal time increasing from 70 to 90 min in Ai9-CCK/DTA animals (Figures 6E,F). Additionally, Ai9-CCK/DTA animals significantly increased average meal size from 0.2 to 0.35 g (Figure 6G) and average meal duration from 5 to 9 min (Figure 6H). In summary, the ablation of CCK-expressing neurons in the VMH region results in a long-term increase in BW, likely due to hyperphagia characterized by lengthier and larger meals occurring less frequently throughout the day. These findings underscore the critical role of CCK^VMH^ neurons in homeostatic energy regulation.

### 3.4 Ablation of CCK^VMH^ neurons leads to hyperglycaemia and glucose intolerance

**Figure 7:**
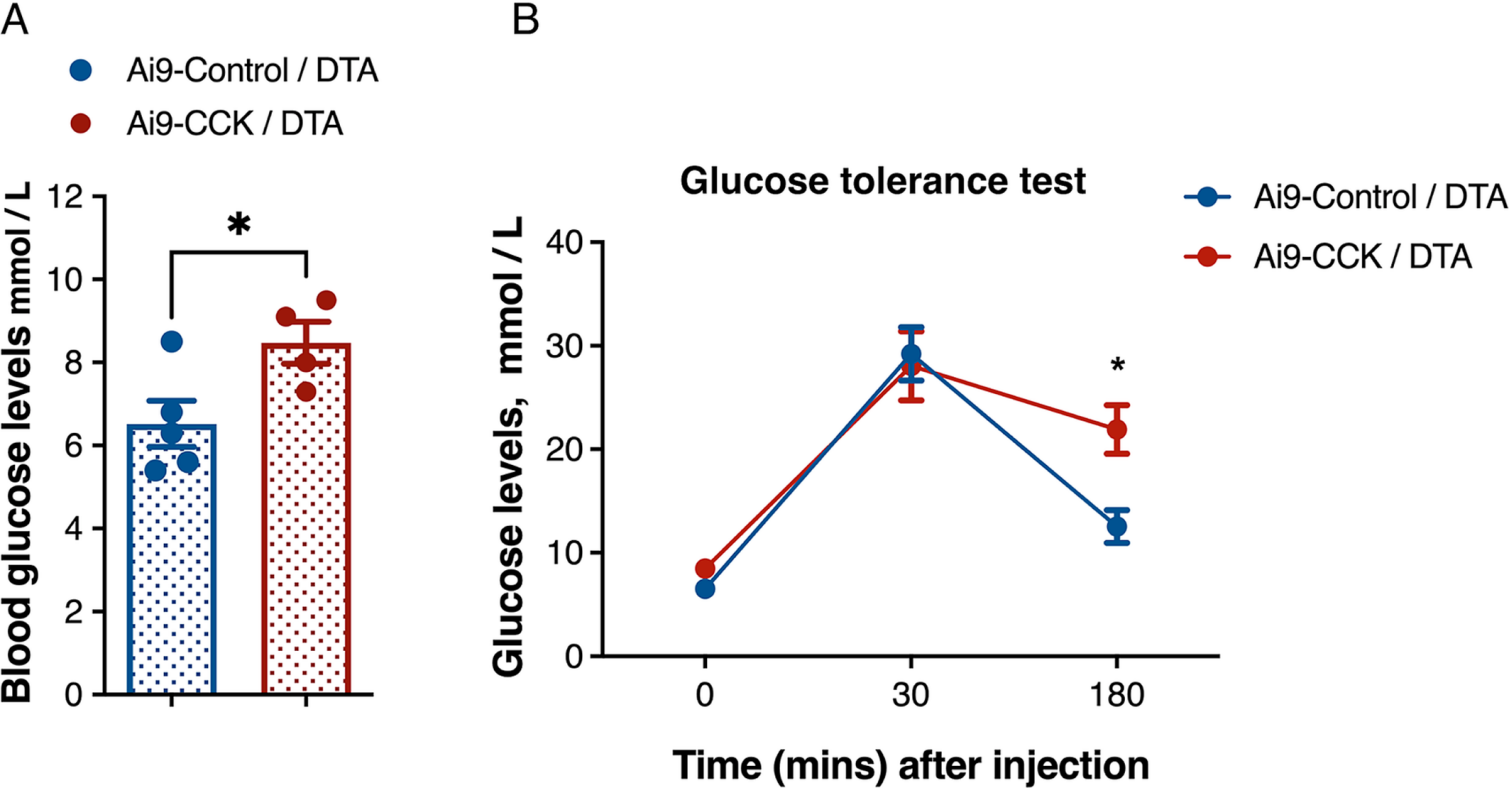
DTA-mediated ablation of CCK^VMH^ neurons results in hyperglycaemia and glucose intolerance. (A) Baseline glucose levels after 16 h fasting before glucose IP injection show that Ai9-CCK/DTA animals are hyperglycaemic (6.520 ± 0.55 vs. 8.475 ± 0.50, *p* = 0.038). (B) The plot shows the GTT of the two groups with a similar increase in blood glucose levels 30 min after IP but glucose intolerance at 180 min *p* = 0.0122. Values are means ± s.e.m. (*n* = 4–5 female mice per group). (A) *p* statistic from unpaired student t-test and (B) from two-way analysis of variance followed by Sidak’s *post hoc* test.

The VMH plays a crucial role in regulating glucose balance and is closely associated with abnormalities in BW and food intake that impact blood glucose control. To explore this further, Ai9-CCK/DTA and Ai9-Control/DTA animals injected with AAV-DIO-DTA underwent a glucose tolerance test (GTT) 3 months post-surgery. Both groups experienced a 16-h overnight fast, beginning at the onset of their active period (6 p.m.), to control for variations in individual food consumption. Blood glucose levels were measured using a handheld whole blood glucometer, with samples taken from the tail vein. Baseline blood glucose levels were recorded the following morning at the start of their resting phase. Ai9-CCK/DTA animals displayed hyperglycaemia, with average glucose levels at 8.5 mmol/L, compared to 6.5 mmol/L in the Ai9-Control/DTA animals (Figure 7A). The animals were given an IP glucose injection for the GTT assay, and their glucose clearance rates were monitored (Figure 7B). Within the first 30 min post-injection, both groups showed a substantial increase in blood glucose levels to 30 mmol/L, with no significant differences observed between the two groups. However, Ai9-CCK/DTA animals maintained elevated blood glucose levels, whereas controls nearly returned to baseline glucose levels within 3 h after the glucose challenge (Figure 7B).

## 4 Discussion

This study provides evidence that CCK-expressing neurons of the VMH play an essential role in energy homeostasis. Acute pharmacogenetic inhibition of CCK^VMH^ neurons leads to hyperphagia by altering food intake and meal patterns. Complete ablation of CCK^VMH^ neurons leads to hyperphagia and, consequently, to obesity and hyperglycaemia.

Due to its essential roles within the hypothalamic nuclei, attempts have been made to identify genes with enriched and distinct regional expression patterns in the VMH (Kurrasch et al., 2007; Liu et al., 2003) and, through gene manipulation, shed light on the distinct role of its heterogeneous cellular composition.

Here, using the HypoMap single-cell gene expression atlas of the murine hypothalamus (Steuernagel et al., 2022) we characterized the heterogeneity of *Cck*-expressing cells within the VMH. Although SF1 is a recognized marker to identify the VMH (Ikeda et al., 1995), it was co-expressed in only about 10% of the CCK^VMH^ cells. Notably, SF1 and CCKBR are predominantly found in the dorsomedial VMH (dmVMH) and central VMH (cVMH) regions, with minimal expression in the vlVMH (Flak et al., 2020; D. W. Kim et al., 2019). This pattern could imply a more substantial presence of CCK in the vlVMH. Yet, our immunostaining assays reveal a more uniform CCK expression across the dmVMH and cVMH, alongside a significant presence in the vlVMH. CCK^VMH^ neurons appear to be a distinct therapeutic target than the broader SF1^VMH^ neurons.

PACAP, on the other hand, is co-expressed in 58% of CCK^VMH^ neurons. Intracerebroventricular administration of PACAP has been shown to decrease food intake, promote leanness in mice (Hawke et al., 2009), and activate sympathetic nerve activity while inhibiting parasympathetic responses (Tanida et al., 2010). Conversely, ablation of PACAP from the VMH or the broader mediobasal hypothalamus results in rapid weight gain, increased adiposity, hyperinsulinemia and hyperglycaemia in mice, with only a slight and delayed rise in food intake (Bozadjieva-Kramer et al., 2021). The loss of PACAP in 58% of CCK^VMH^ neurons may partially explain the observed increases in BW and hyperglycaemia following CCK^VMH^ ablation.

Somatostatin is co-expressed in approximately 36% of CCK^VMH^ neurons and is crucial in regulating satiety and obesity factors both centrally and peripherally (Kumar and Singh, 2020). Somatostatin was found to have both orexigenic actions, for example, through inhibition of CCK (Herzig et al., 1994) and action on the somatostatin receptor 2 inducing feeding and drinking behavior (Stengel et al., 2010, 2015), and anorexigenic action, for example, when administered peripherally (Bray, 1995). Loss or acute inhibition of the CCK^VMH^ neurons expressing SST may contribute to the changes in the feeding behavior observed in our pharmacogenetic mouse model.

CCKBR is the primary CCK receptor in the brain (Flak et al., 2020) and is moderately expressed throughout the VMH, with low expression in the vlVMH. Moreover, CCKBR is found in three times as many VMH cells as CCK, indicating minimal autocrine effects of CCK. CCKBR^VMH^ neurons specifically raise the blood glucose setpoint without affecting energy expenditure and BW, both under normal conditions and during the counterregulatory response (CRR). They do so by regulating hepatic glucose production independently of islet hormones, highlighting the brain’s central role in glucose homeostasis (Flak et al., 2020). When CCKBR is knocked out systemically in mice, it leads to obesity characterized by increased food intake and fat accumulation due to adipocyte hypertrophy. This also disrupts glucose regulation, causing elevated fasting blood glucose and insulin levels, impaired glucose tolerance, and hepatic insulin resistance (Clerc et al., 2007). In contrast, the silencing of CCKBR^VMH^ neurons results in hypoglycaemia (Flak et al., 2020). In our study, acute inhibition of CCK^VMH^ neurons leads to hyperphagia, and complete ablation of these neurons leads to hyperphagia-associated obesity and hyperglycaemia. This information and the slight overlap between the two subpopulations expressing CCK and the CCKBR in the VMH suggest that they have distinct functions within their circuits. This emphasizes that CCK can still affect the CCKBR^VMH^ neurons by being produced by other cells in the hypothalamus or from the periphery.

In this study, we utilised two approaches to investigate the role of CCK-expressing cells in the VMH. The first approach involved spatiotemporal control of the activity of CCK^VMH^-specific cells using chemogenetic cellular inhibition (hM4Di). The second approach involved ablating CCK^VMH^ cells using DTA viral transduction, which aimed to remove the cells expressing CCK in the VMH, potentially resulting in a more pronounced phenotype.

For the chemogenetic approach, incremental doses of CNO revealed a dose-dependent increase in food intake, with significant effects observed at 3 mg/kg BW. This dose-dependent response indicated that higher CNO concentrations effectively inhibited sufficient CCK^VMH^ neurons to modulate food intake. This experiment revealed that CCK^VMH^ neurons significantly influence food intake and meal patterns. The increase in daily food intake and meal frequency following hM4Di activation suggests that CCK^VMH^ neurons play a critical role in suppressing feeding behavior. This finding is consistent with the known anorexigenic effects of CCK in peripheral tissues (Ahn et al., 2022; Cawthon and de La Serre, 2021; Gibbs et al., 1973; Steinert et al., 2017), extending these effects to central mechanisms within the hypothalamus. Interestingly, the increased food intake manifested as a delayed response within the first 6 h post-injection. This delay may suggest that the CCK^VMH^ neurons are involved in a broader neural circuit activation that regulates feeding behavior, supporting a delay in phenotype manifestation.

To assess changes in food intake resulting from the inhibition of CCK^VMH^ neurons, we analyzed various meal patterns, including meal frequency, intermeal intervals, meal size, and duration. Meal frequency increased 6 h after CNO injection without affecting total food intake. The intermeal time significantly decreased with the rise in meal frequency and number of meals. Given that meal duration remained unchanged, it suggests that the increase in meal frequency was temporarily offset by a slight reduction in meal size, which may explain the unchanged cumulative food intake 6 h post-inhibition. Prolonged activation of the hM4Di and the associated GPCR signaling pathways could have long-lasting, cell-type-specific effects. Inhibition of CCK^VMH^ neurons indeed led to a sustained increase in meal frequency throughout the day. In the 24 h data analysis, meal sizes did not change, indicating that the initial compensatory mechanism in response to increased meal frequency was overridden by continued inhibition. Other components of the satiety neuronal circuitry might fine-tune the VMH neuronal circuit’s sensitivity, including CCK-expressing neurons. In summary, inhibition of CCK^VMH^-expressing neurons increases meal frequency, with meal size initially adjusted to prevent excessive energy intake. However, chronic inhibition eliminates this adjustment, increasing total food intake due to higher meal frequency. These results reveal a potential modulatory window for intervention considering the delay in phenotype manifestation.

Meanwhile, the ablation of CCK^VMH^ neurons led to a 28% increase in BW compared to controls at 14 weeks post AAV-DIO-DTA injection. The obese phenotype emerged about a month post-injection, corresponding with the typical time frame for optimal AAV expression and complete neuronal ablation. The obesity resulted from hyperphagia, with larger meal sizes and longer durations. Interestingly, meal numbers decreased, and intermeal intervals increased significantly without CCK^VMH^ neurons, suggesting dysregulated compensatory mechanisms attempting to balance increased food intake with larger, longer meals. The energy surplus might also heighten peripheral satiety signals, activating hindbrain inhibitory circuits.

The dysregulation of food intake caused by CCK^VMH^ neuron ablation differs from acute inhibition using hM4Di. DTA ablation nearly eliminates all CCK-positive neurons in the VMH, disconnecting them from local circuits. During hM4Di-mediated inhibition, neurons remain intact and continue to receive presynaptic inputs, possibly engaging in satiety signal processing (for example, glucose, leptin/insulin, ghrelin) that do not require synaptic neurotransmission, which is inhibited by hM4Di. The inhibition by hM4Di is ligand-dependent, creating a temporal inhibition window, varying based on the injection schedule. Thus, CCK^VMH^ neurons likely modulate food intake through multiple mechanisms. Inhibition of neurotransmission increases meal frequency, suggesting postsynaptic partners contribute to satiety signals by adjusting energy intake per meal. Without CCK input, meal consumption becomes disrupted, initially decreasing meal size, which normalizes over time, though meal onset becomes more frequent. Conversely, neuron ablation results in prolonged meals with increased size, indicating a role in integrating neurohormonal satiety signals independent of synaptic communication. Increased meal sizes might trigger compensatory satiety circuits from the gastrointestinal tract and vagal pathways to the brain, ultimately reducing meal frequency.

Given the VMH’s role in glucose homeostasis and CRR, we found that CCK^VMH^ ablation caused hyperglycaemia, with baseline blood glucose levels higher than controls after 16 h of fasting. These animals also showed glucose intolerance, maintaining higher blood glucose levels up to 3 h post-glucose administration. These findings indicate that CCK-expressing neurons contribute to glucose sensing and homeostasis within the VMH, and their absence results in sustained high blood glucose levels.

As hormone-based obesity treatments gain prominence, GLP-1 receptor agonists have established themselves as frontrunners in this rapidly advancing field (Müller et al., 2019). While both GLP-1 and CCK have relatively short half-lives (Skibicka and Dickson, 2013), their pharmacological receptor agonists exhibit significantly prolonged effects (Christoffersen et al., 2020; Goldenberg and Steen, 2019). In pursuing more effective therapies for obesity and diabetes (Rodriguez et al., 2023), CCK analog NN9056 has emerged as a promising candidate, especially when combined with the GLP-1R agonist semaglutide (Zhou et al., 2024). Although peripheral CCK action is vital for regulating food intake and energy balance, our findings demonstrate that CCK-expressing neurons in the VMH play a pivotal role in controlling feeding behavior and influencing glucose homeostasis by integrating neurohormonal satiety signals and synaptic inputs. Understanding the neural circuits involving CCK^VMH^ neurons may lead to more targeted and effective therapies in this crucial area.

## Data Availability

The datasets presented in this study can be found in the article, source data and Supplementary material. Further inquiries can be directed to the corresponding author.
