# Supplementary material for: Cholecystokinin-expressing neurons of the ventromedial hypothalamic nucleus control energy homeostasis
Source: Front Cell Neurosci. 2024 Oct 28;18:1483368. doi: 10.3389/fncel.2024.1483368 (PMC11550940; doi:10.3389/fncel.2024.1483368)
Supplement: Supplementary file 2 [file Data_Sheet_1.PDF]

1 Supplementary Figures

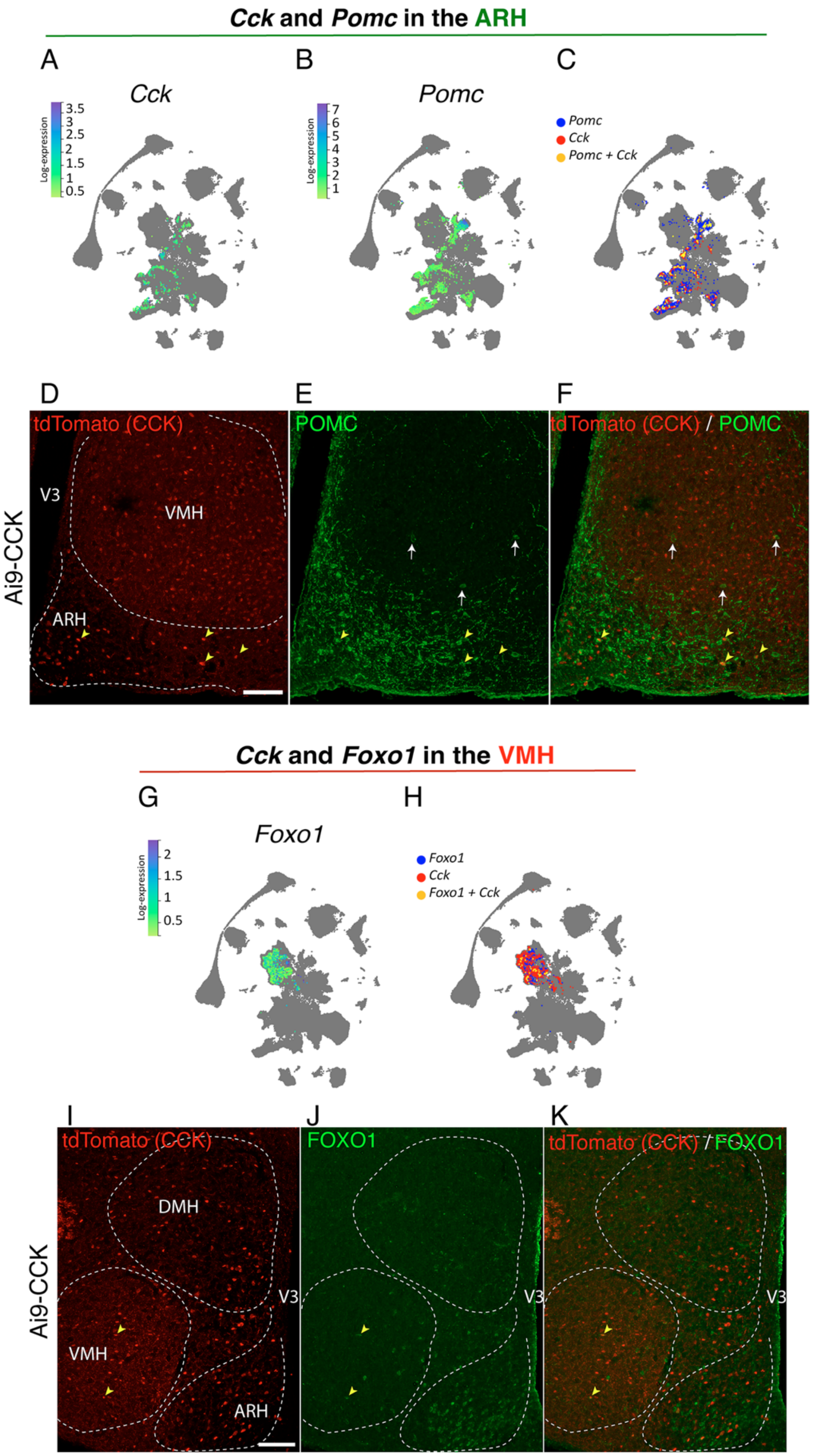

Supplementary Figure 1. Specificity of the BAC-CCK-Cre expression pattern in the VMH and ARH of Ai9-CCK.

(A-B and **G)** HypoMap (Steuernagel et al., 2022) UMAP expression of *Cck* and *Pomc* in the ARH and *Foxo1* in the VMH, respectively. A specific selection of positive expression values in the ARH and VMH regions of 2-month-old mice is shown. The sidebar corresponds to log-normalized expression values scaled to the maximum of each gene, with the rest of the hypothalamus in grey. **(C** and **H)**, HypoMap (Steuernagel et al., 2022) UMAP of cells showing co-expression of *Cck* in red, and either *Pomc* (C) or *Foxo1*(H) in blue and co-expressing cells in yellow. Only positive expression values are shown. **(D-F)** Representative sections through the hypothalamus of Ai9-CCK strain showing CCK+tdTomato expression pattern in the VMH and ARH (D), POMC immunofluorescence (E) and a colocalisation (F) where it can be appreciated that very few POMC positive cells colocalise with tdTomato in the ARH, and very few POMC positive cells are visible in the VMH. **(I-K)** Representative sections through the hypothalamus of Ai9-CCK strain showing CCK-tdTomato expression pattern in the VMH and ARH (I), FOXO1 immunofluorescence (J) and a colocalisation (K) where very minimal overlap can be appreciated between FOXO1 positive cells and CCK+tdTomato in the ARH and VMH, as expected. The white arrow indicates no colocalisation, while the yellow arrowhead indicates colocalisation. Panels (D, I), scale bar 100 μm.

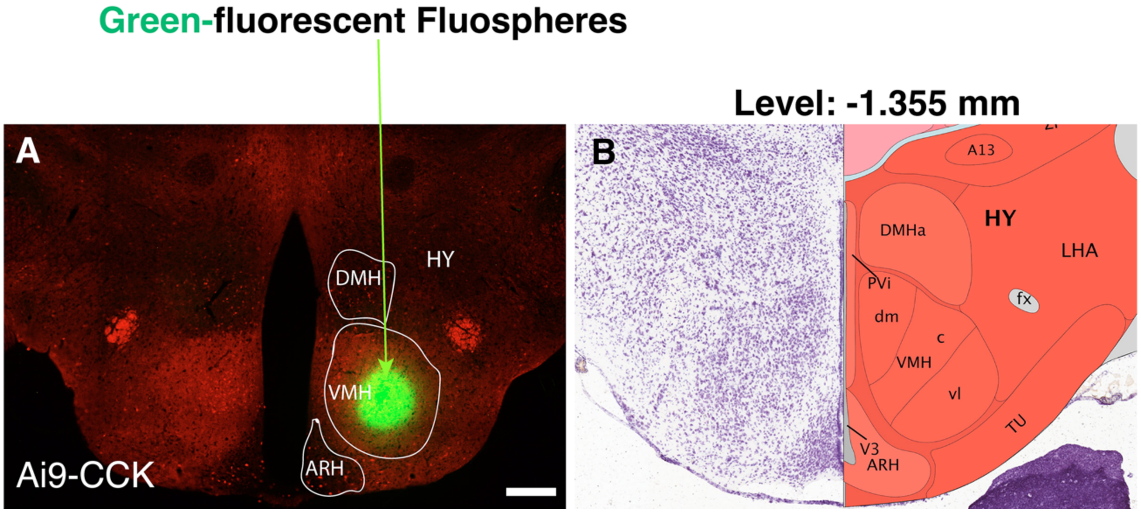

Supplementary Figure 2. Stereotactic delivery of green-fluorescent latex particles in the hypothalamic VMH region of Ai9-CCK mice.

(A) CCK^{VHM} coordinates were successfully established by injecting 200 nl of 0.1% w/v latex particles (0.04 µm diameter, Invitrogen, cat no: F-8795) unilaterally at the stereotactic coordinates from bregma -1.155 mm to -1.355 mm (the latter is shown above in A). **(B)** Tile images of 20 µm thick coronal sections. Image and respective atlas reference, emphasising the hypothalamic region showing green fluorescence exclusively in the VMH; atlas reference images (from ©2014 Allen Institute for Brain Science, Allen Mouse Brain Atlas [Internet]). Scale bar panel A, 250 µm.

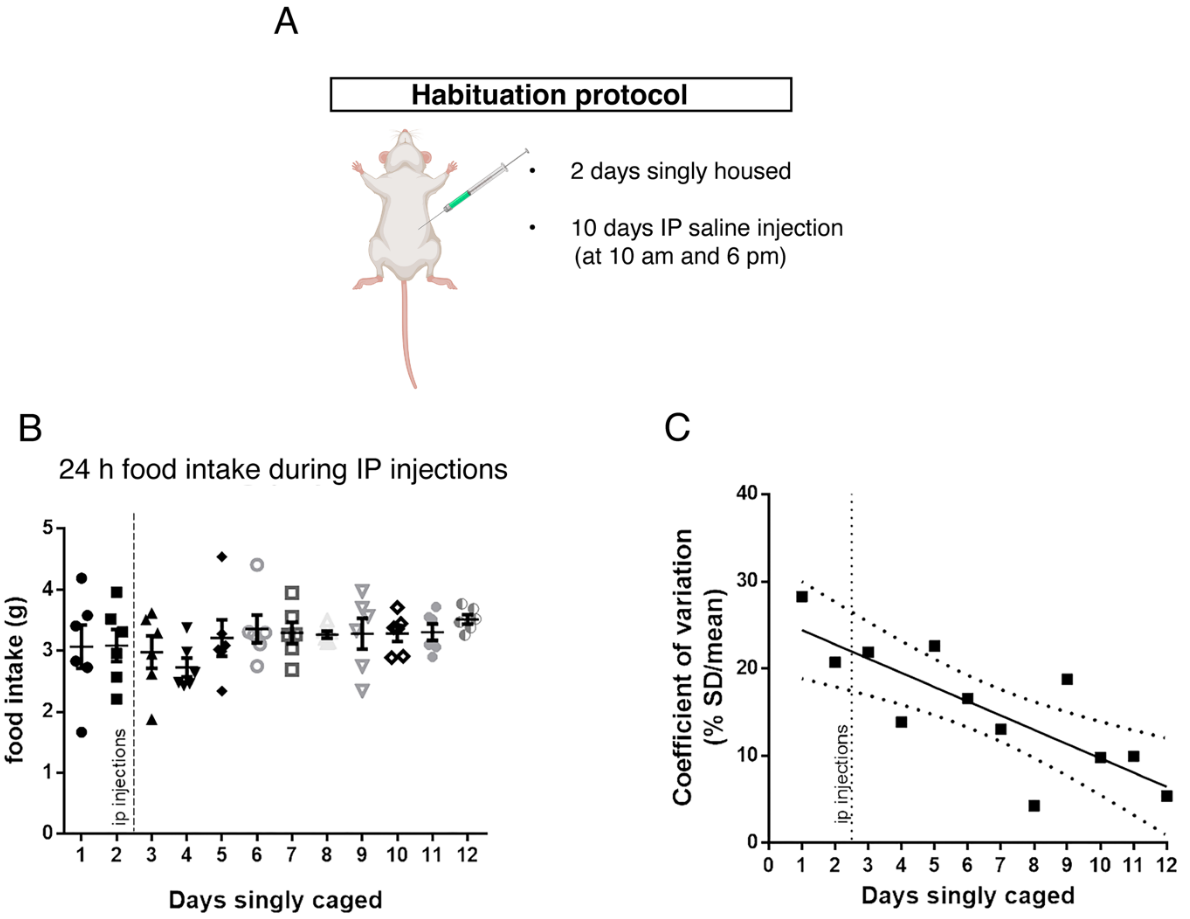

Supplementary figure 3. Establishing a daily administration protocol by intraperitoneal injection (IP) to monitor food intake.

(A) Protocol to assess food intake and meal patterns. Animals were singly housed for two days and then habituated for ten days with IP saline injections twice daily (10 a.m. and 6 p.m.). **(B)** Twenty-four-hour food intake of BAC-CCK-Cre animals two days before and ten days after receiving IP saline injections. In the first two days of a single housing, the animals consumed, on average, 3.078 ± 0.307 g of pellets cumulatively throughout the day. Still, the variation within the group was high due to the change in their environment. In the first three days of saline injections (3-5), the measurements fluctuated but remained stable throughout, with food intake equal to 3.368 ± 0.114 g in the last three days of saline IP. **(C)** The protocol for habituation is sufficient to reduce variability in the feeding data, as revealed by the coefficient of variation of their daily food intake before and after the saline IP injections (n = 6 mice, males). Image in A was created with BioRender.com.

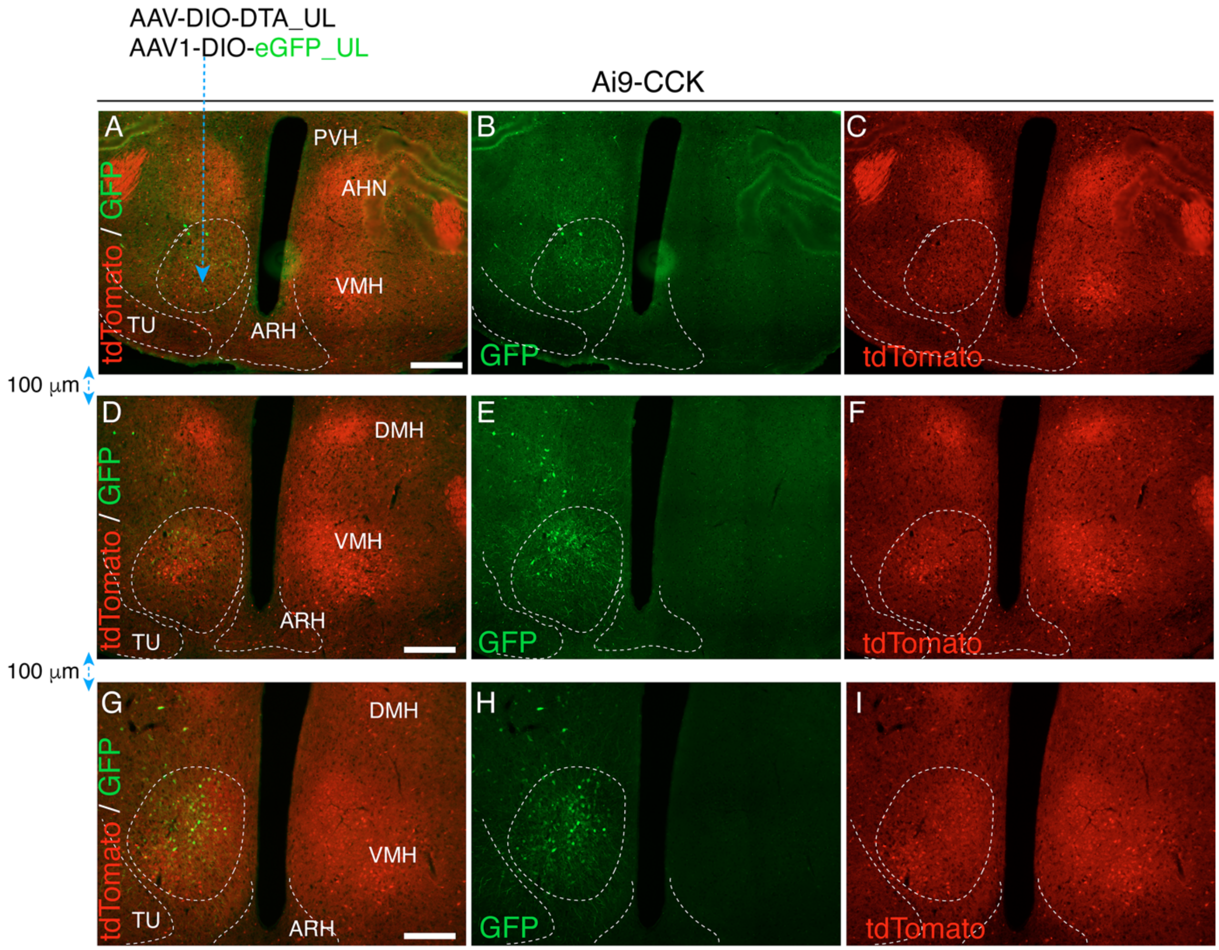

Supplementary Figure 4. AAV-DIO-DTA deletes VMH CCK+tdTomato cells without affecting non-transduced surrounding cells.

(A, D, G) Coronal sections highlighting the hypothalamus of an Ai9-CCK mouse showing a unilateral injection of AAV-DIO-DTA together with AAV1-DIO-eGFP in the VMH. Mice were injected with the established concentration of DTA particles (at 2.32 x 10^{11} GC/ml) and 50 nl of AAV1-DIO-eGFP to help identify the injection site and the extent of CCK ablation in the VMH. **(A)** A significant reduction of CCK+tdTomato cells is evident on the injected side. **(B)** It shows the VMH injected side by the GFP where only some GFP are still evident, as are some CCK+tdTomato cells seen in panel **(C)**. **(D-F and G-I)** Partial ablation of CCK+tdTomato cells co-expressing eGFP is instead observed in sections 100 and 200 µm away from the injection site **(A-C)** in the posterior direction, respectively. Higher numbers of CCK+tdTomato cells colocalising with eGFP are evident in panels **(D** and **G)** relative to the distance from the injected site. UL, unilateral; VMH, ventromedial hypothalamus; DMH, dorsomedial nucleus; ARH, arcuate hypothalamic nucleus; TU, tuberal nucleus. Scale bars panel A, D, G, 250 µm.

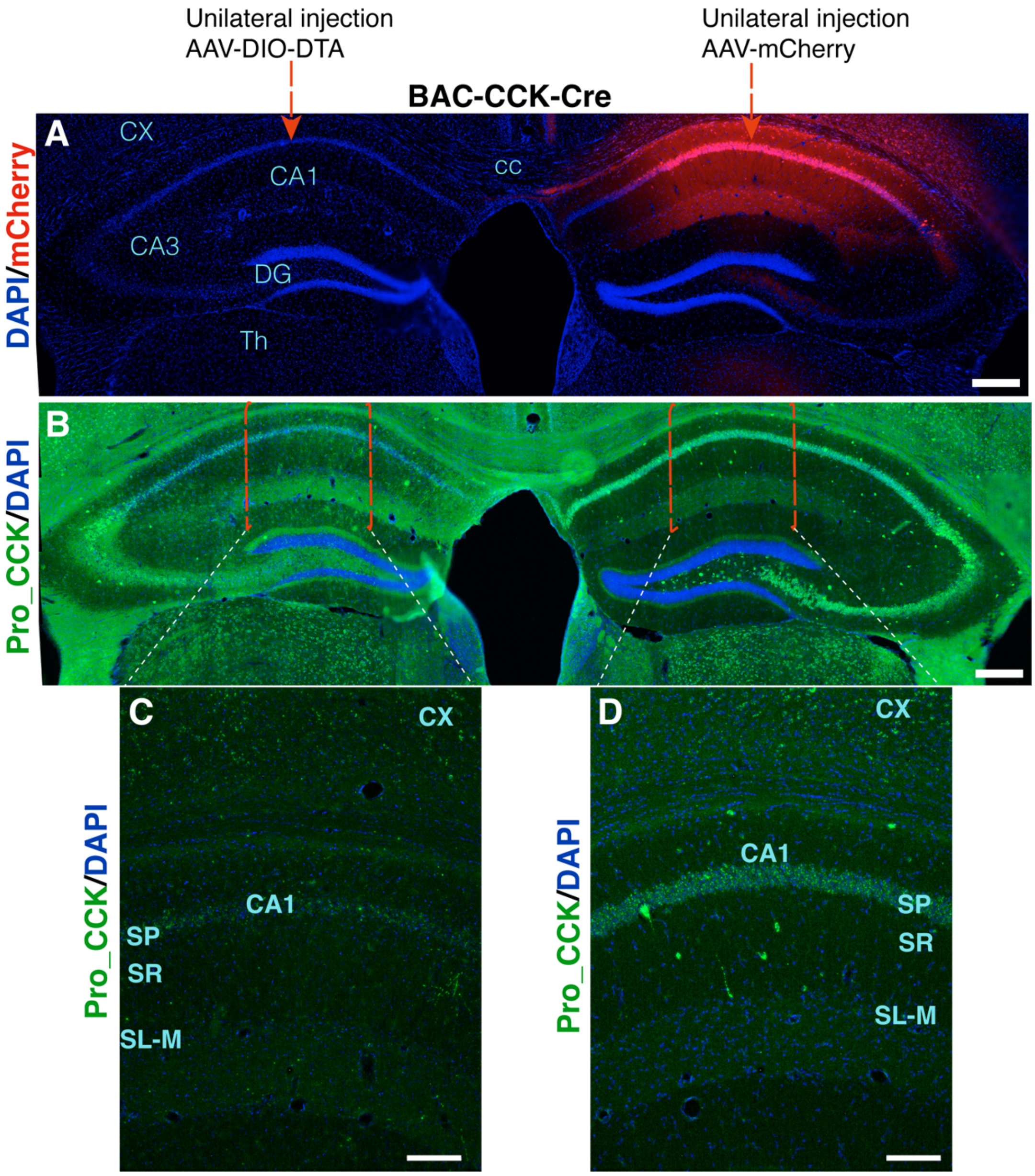

Supplementary Figure 5. The specificity of the BAC-CCK-Cre line and the DTA ablation.

(A) Intracranial injection of the AAV-DIO-DTA in the right hemisphere and an AAV-mCherry in the left hemisphere at the CA1 region level. **(B)** Immunohistochemistry of coronal sections with a-proCCK antibody revealed loss of staining on the DTA injected hemisphere at the CA1 region and no loss of CCK expression on the other hemisphere where AAV-mCherry particles were injected or of surrounding CCK-neurons such as those in the CA3 region or thalamus. **(C-D)** Higher magnification of the CA1 injected region shows no CCK staining in (C) compared to panel (D). CA, cornu Ammonis; cc, corpus callosum; CX, cerebral cortex; DG, dentate gyrus; SL-M, stratum lacunosum-moleculare; SP, stratum pyramidale; SR, stratum radiatum; Th, thalamus. Scale bars panel A-B, 250 µm; C-D, 100 µm.
